# The Impact of Immune Interventions: A Systems Biology Strategy for Predicting Adverse and Beneficial Immune Effects

**DOI:** 10.3389/fimmu.2019.00231

**Published:** 2019-02-15

**Authors:** Marjolein Meijerink, Tim van den Broek, Remon Dulos, Lotte Neergaard Jacobsen, Anne Staudt Kvistgaard, Jossie Garthoff, Léon Knippels, Karen Knipping, Geert Houben, Lars Verschuren, Jolanda van Bilsen

**Affiliations:** ^1^TNO, Zeist, Netherlands; ^2^Arla Foods Ingredients, Aarhus, Denmark; ^3^Danone Food Safety Center, Utrecht, Netherlands; ^4^Danone Nutricia Research, Utrecht, Netherlands; ^5^Utrecht Institute of Pharmaceutical Sciences, Utrecht University, Utrecht, Netherlands

**Keywords:** biomarkers, safety assessment, systems biology, immune intervention, network databases

## Abstract

Despite scientific advances it remains difficult to predict the risk and benefit balance of immune interventions. Since a few years, network models have been built based on comprehensive datasets at multiple molecular/cellular levels (genes, gene products, metabolic intermediates, macromolecules, cells) to illuminate functional and structural relationships. Here we used a systems biology approach to identify key immune pathways involved in immune health endpoints and rank crucial candidate biomarkers to predict adverse and beneficial effects of nutritional immune interventions. First, a literature search was performed to select the molecular and cellular dynamics involved in hypersensitivity, autoimmunity and resistance to infection and cancer. Thereafter, molecular interaction between molecules and immune health endpoints was defined by connecting their relations by using database information. MeSH terms related to the immune health endpoints were selected resulting in the following selection: hypersensitivity (D006967: 184 genes), autoimmunity (D001327: 564 genes), infection (parasitic, bacterial, fungal and viral: 357 genes), and cancer (D009369: 3173 genes). In addition, a sequence of key processes was determined using Gene Ontology which drives the development of immune health disturbances resulting in the following selection: hypersensitivity (164 processes), autoimmunity (203 processes), infection (187 processes), and cancer (309 processes). Finally, an evaluation of the genes for each of the immune health endpoints was performed, which indicated that many genes played a role in multiple immune health endpoints, but also unique genes were observed for each immune health endpoint. This approach helps to build a screening/prediction tool which indicates the interaction of chemicals or food substances with immune health endpoint-related genes and suggests candidate biomarkers to evaluate risks and benefits. Several anti-cancer drugs and omega 3 fatty acids were evaluated as *in silico* test cases. To conclude, here we provide a systems biology approach to identify genes/molecules and their interaction with immune related disorders. Our examples illustrate that the prediction with our systems biology approach is promising and can be used to find both negatively and positively correlated interactions. This enables identification of candidate biomarkers to monitor safety and efficacy of therapeutic immune interventions.

## Introduction

The immune system plays a crucial role in maintaining health, and hence immune disturbances play a key role in immunodeficiency, infectious diseases, cancer, obesity associated pathology, geriatrics, allergy, autoimmunity, mental disorders, and toxicity in general. Advances in our understanding of immune-related disorders has led to the development and improvements of immunotherapy of these diseases such as atopic diseases, autoimmunity, and tumor immunity. In atopic diseases, non-specific [e.g., anti-IgE (1)] and allergen-specific immunotherapies (2–4), have been used successfully for a wide range of allergies, even though to date the allergen-specific immunotherapies aimed at inducing permanent tolerance in food allergies seem limited. Besides “classical” pharma, biopharmaceuticals (e.g., type I interferons, interleukin-2) have been used widely to restore immune disbalances in therapy of cancer or inflammatory and autoimmune diseases (5).

The advances in our understanding of immune-related disorders and possible therapeutic approaches, coincide with the awareness that the restoring of immune balances might also induce adverse events. Effective tumor immunity requires the induction of the same responses that underlie autoimmunity, whereas autoimmunity is driven by dysregulation of the same mechanisms that are involved in host defense and immune surveillance. Therefore, as we manipulate the immune system to treat cancer or autoimmunity, we inevitably unbalance the vital mechanisms that regulate self-tolerance and antimicrobial resistance (6). Also, a repeated injection of interferon (IFN)-β which is used in cancer therapy can result in a break in immune tolerance to self-antigens (7), and might result in the induction of autoimmune responses, as has been reviewed by Toomer and Chen (8). Azacitidine (Vidaza) is a hypomethylating agent, which has been successfully used to treat acute myeloid leukemia and myelodysplastic syndromes (MDS) (9). However, infectious events, specifically bacterial infections, are one of the most frequent complications during therapy with azacytidine (10). Retuximab, a monoclonal anti-CD20 antibody, is used to treat B-cell non-Hodgkin lymphoma, B-cell leukemia, and rheumatoid arthritis. Its side effects include infections and fever or even more seriously, the reactivation of infections.

These examples illustrate the risks and benefits of restoring the balance in immune-related disorders by novel treatment strategies. Therefore, as the number of immunotherapeutic options increase, better knowledge of immune pathways is needed to improve the ability to tread the fine line between treatment efficacy and unacceptable adverse events. The same holds true with respect to (the development of) approaches for the prevention of immune (related) diseases.

Even though several starting points for immune health interventions have been identified and are being developed into prophylactic or therapeutic approaches, there is no regulatory guidance about how to assess the risk and benefit balance of such interventions. This particularly applies to the safety assessment of (nutritional) immune interventions in early life when young children often are considered most vulnerable. Regulatory authorities (EFSA, JECFA) stress the necessity to address the safety assessment of new product applications. At the moment final safety confirmation comes from expensive and lengthy clinical follow up studies. Therefore, there is a clear need for a science-based approach to assess the safety and benefit of (nutritional) immune interventions.

Recent technological advances have made it possible to collect comprehensive datasets at multiple molecular and cellular levels (genes, gene products, metabolic intermediates, macromolecules, cells). Over the past years, biologists have increasingly invested in building network models to illuminate the functional and/or structural relationships of these components into coherent frameworks, thereby also helping to provide insight into how disturbances of the cellular or organismal environment lead to changes in cell or organism behavior.

**Figure 1:**
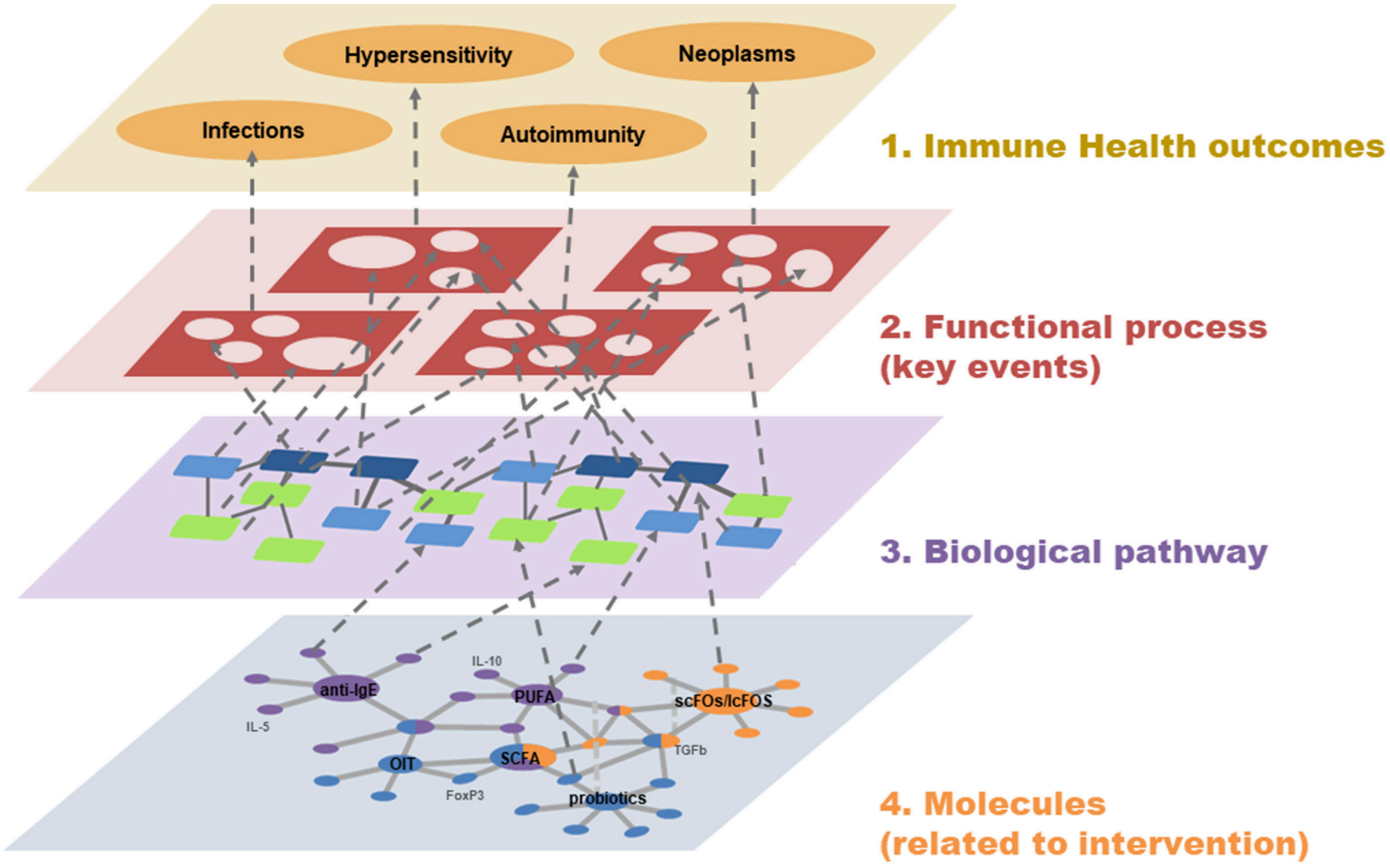
A systems biology view on Immune Health. Four interacting layers are used to demonstrate the relationships between Immune Health (top level) and molecules/biomarkers (bottom level, mock example). The two middle layers represent the involved functional processes (key events) and biological pathways (cascade of molecule-molecule interaction) that connect Immune Health with the related molecules and biomarkers.

So far, most studies or research groups have collected big data sets from several –omics-platforms to understand the bigger picture by putting the pieces together. Despite enormous amounts of resources spent on this approach, the number of useful and practical biomarkers remains disappointing. Therefore, we aimed to follow a deductive approach by first defining which immune health effects are crucial to be monitored in terms of efficacy/safety. This was followed by the identification of key immune pathways involved in those immune health end points, and subsequently the selection and ranking of those key markers/parameters that are linked to the immune pathways. Instead of gathering large amounts of novel–omics data, we will start at the most important feature in the top layer of immune health (Figure 1) and ultimately select the candidate markers at the bottom layer that are useful for predicting/monitoring the efficacy/safety of immune interventions.

The aim of this paper is to illustrate how a systems biology approach can help identify key immune pathways involved in those immune health end points and select crucial biomarkers to monitor effects upon immune interventions. In this paper we will focus on immune health end points *Hypersensitivity, Autoimmunity* and resistance to *Infection and Cancer* and highlight the effects of immune intervention on these end points.

## Materials and Methods

### Brief Overview of Major Immune Health End Points

An inventory of the available literature regarding different health end points was performed using the databases Scopus and PubMed. A well-balanced immune system is key for overall health and well-being. The immune system maintains homeostasis by mounting non-specific innate and specific adaptive responses to threats of (changing) microbiological exposure. At the same time, the immune system should have a tolerant response to self-antigens and harmless non-self-antigens and allergens, as an inappropriate response to such antigens contributes to different immune health end points. For example, an inappropriate or exaggerated immune response to allergens may lead to hypersensitivity type I reactions, which include allergic rhinitis (hay fever), allergic asthma, atopic dermatitis (eczema), and food allergy. In addition, sustained responses to persistent antigens, such as autoantigens or those derived from commensal micro-organisms, lead to tissue remodeling and disturbance of function of the affected tissue and to diseases such as rheumatoid arthritis, inflammatory bowel disease, and psoriasis. Lastly, the immune system plays an important role in suppressing tumor development and shaping tumor immunogenicity.

Therefore, in this manuscript we focused on the following immune health endpoints immune health end points *Hypersensitivity, Autoimmunity* and resistance to *Infection and Cancer* and highlight the effects of immune interventions on these end points.

### Key Mechanistic Processes Driving the Four Health Endpoints

There is a considerable amount of *in vivo* and *in vitro* data available describing molecular and cellular events potentially involved in hypersensitivity, autoimmunity and resistance to infection and cancer. These events can be organized in a sequence of related events that could plausibly lead to a certain health endpoint. During the literature study, for each of the four health endpoints, these molecular and cellular events were described in a framework. These proposed frameworks simplify complex biological process by collecting, organizing, and evaluating data that describe the events at a biological level of organization with relevance for risk assessment. The application of these frameworks allows us to identify the major molecular initiating key processes underlying hypersensitivity, autoimmunity and resistance to infection and cancer. The processes included in those frameworks are still highly complex at molecular-cellular level, so the challenge is to integrate the processes to better understand the mechanistic pathways. These frameworks offer the opportunity to select crucial biomarkers to monitor effects upon nutritional immune interventions.

### Connecting Key Mechanistic Processes to Candidate Biomarkers

The key mechanistic processes in the defined frameworks were used to mine the Gene Ontology database and the Comparative Toxicogenomics Database (CTD) to select pathways and/or genes related to immune health endpoints as identified above. Knowledge used to generate CTD and GO database involves information from both clinical/medical and pre-clinical settings. The Gene Onthology Consortium defines concepts related to gene functions (“GO terms”), and how these functions relate to each other (“relations”) (www.geneontology.org/). These GO terms enable us to connect genes/proteins to diseases using GO identification numbers and MeSH identification numbers. Neo4J is a graph-database with query-based calculations (Neo4j, Inc., San Mateo, CA, USA) and is used, together with Venn-diagrams (11) to visualize and calculate the unique and overlapping genes/proteins among the health endpoints/processes. These sets of genes/proteins form the basis for a rank-based selection of candidate biomarkers for these key mechanistic processes.

### Proof of Principle/*in silico* Test Cases

After selecting a large set of genes for each immune health endpoint and predicting their role in the initiation in disturbed immune functions, the next step was to check whether this set of genes could be validated. This was done by predicting whether an immune intervention can result in a higher susceptibility to any of the health endpoint disturbances. To this end, a set of chemicals and food substances were selected that are currently used as immune interventions of which (i) adverse/beneficial effects are described and (ii) top interacting genes are described in the CTD. The CTD contains curated data on the top interacting genes affected by a chemical/food substance (http://ctdbase.org/help/ixnQueryHelp.jsp). Using these curated data, the top interacting genes were compared with our previously identified set of genes predicted to be involved in the initiation of immune disorders, to check whether the previously described adverse/beneficial effects confirmed our prediction.

## Results and Discussion

### Key Processes Driving the Four Immune Health Endpoints

In order to select molecules/genes involved in immune health disturbances, overviews were generated of all key processes likely to be involved in the induction of hypersensitivity (which can be divided in four different types: hypersensitivity type I, II, III, IV), autoimmunity, and resistance to infection and cancer (Supplementary Figures 1–7) (12–19). These key processes are described in the legends and visualized in biological frameworks. These processes were used to select pathways/genes.

It must be noted that an appropriately functioning immune system is a dynamic system in which everything is kept in an optimal balance. So even though this balance might be disturbed by the upregulation/activation of certain pathways/molecules, this does not automatically result in a malfunctioning of the immune system, as a healthy dynamic immune system will eventually return to a balanced state. Therefore, it is of importance that the predicted biomarkers that might influence the immune functions after immune intervention, are validated in an appropriate assay/study.

### Connecting Key Mechanistic Processes to Candidate Biomarkers

**Figure 2:**
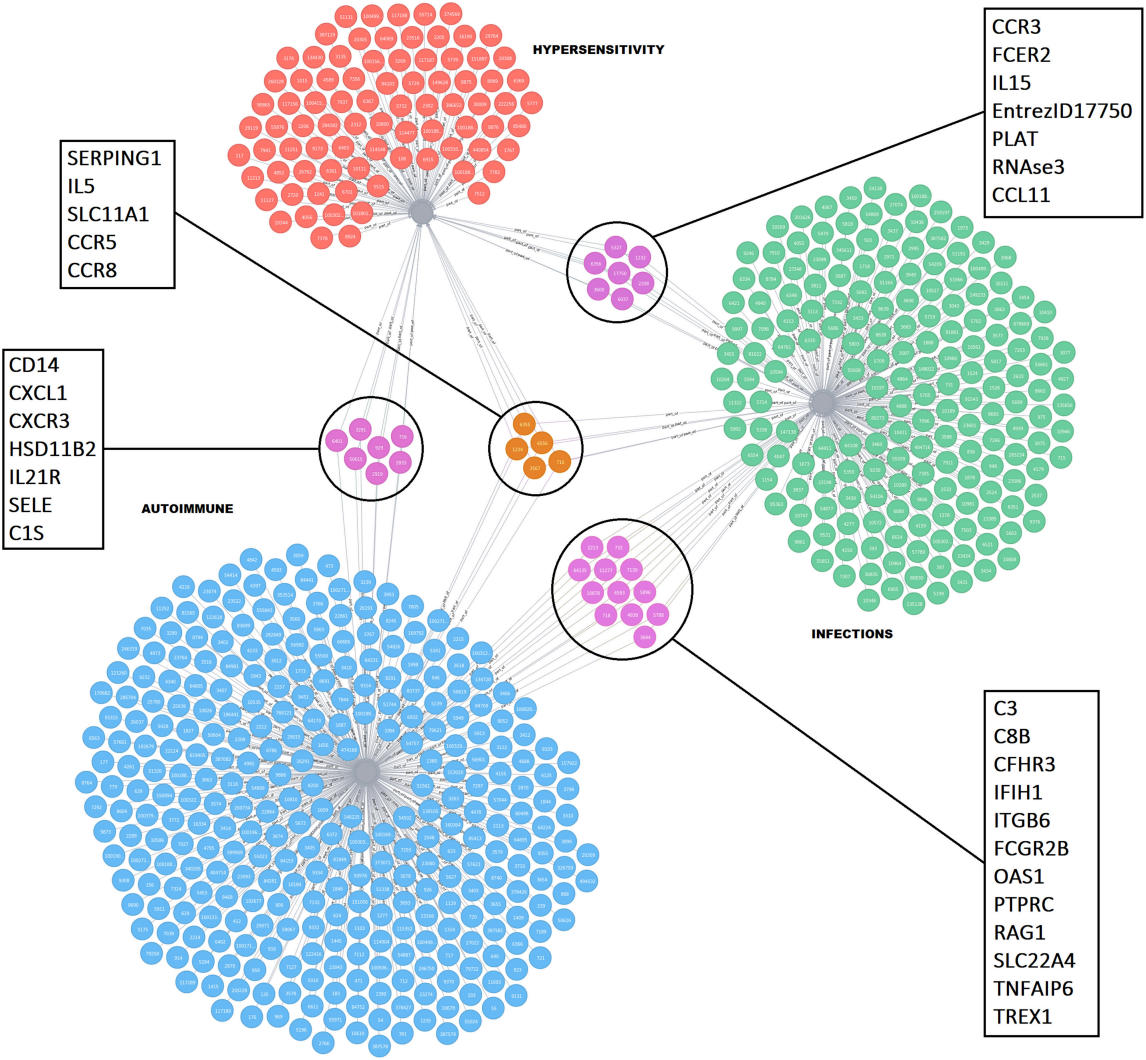
Network visualization showing unique and shared molecules between hypersensitivity, infections, and autoimmune disorders. The red nodes indicate molecules related to hypersensitivity, blue nodes indicate molecules related to autoimmune, and green nodes indicate molecules related to infections. Molecules in the highlighted circles are shared between the indicated immune health end points. The squares indicate the names of the shared molecules. The molecules confined to the fourth immune endpoint “cancer” are not depicted for clarity reasons as they are highly shared among the other immune health endpoints.

**Figure 3:**
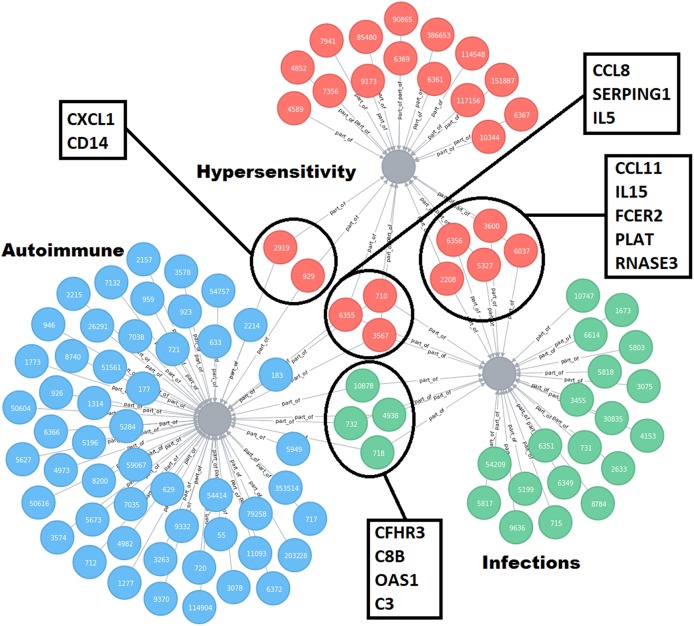
Refined network visualization of the unique and shared molecules between hypersensitivity, infections and autoimmune disorders, and predicted to be secreted in plasma. All molecules labeled to be secretable according UniProt database are included in the network. The red nodes indicate molecules related to hypersensitivity, bleu nodes indicate molecules related to autoimmune, and green nodes indicate molecules related to infections. Molecules in the highlighted circles are shared between the indicated immune health end points. The squares indicate the names of the shared molecules.

**Figure 4:**
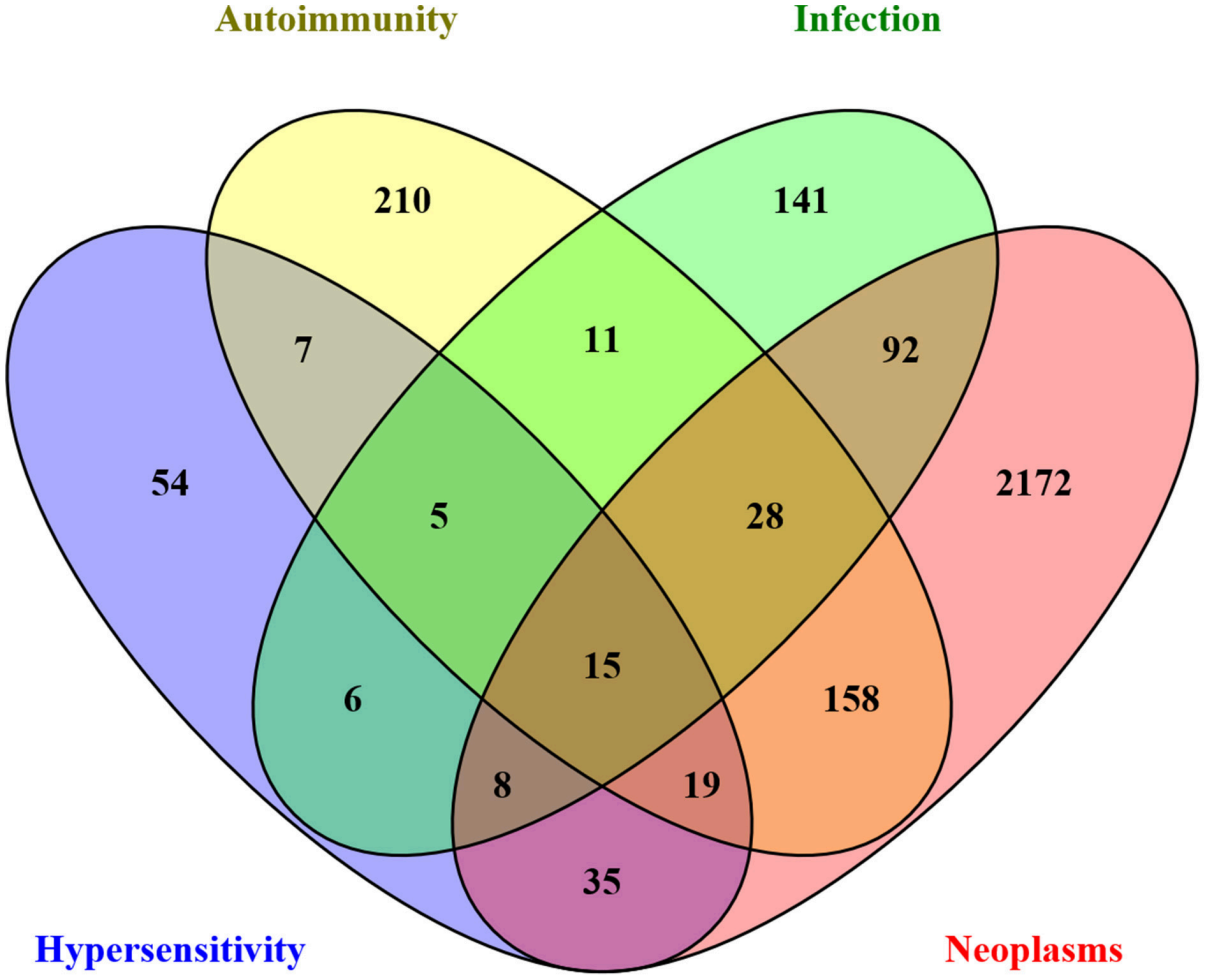
Venn diagram illustrating the (shared) sets of molecules involved in key mechanistic processes driving immune health endpoint disturbances. The key mechanistic processes were used to mine the GO database and CTD to select the depicted number of genes.

The key mechanistic processes were used to mine the GO database and CTD to select pathways and/or genes driving the four immune health endpoints as identified above. In the CTD the molecules were collected related to each immune health endpoint: hypersensitivity (hypersensitivity D006967: total 184 molecules), autoimmunity (autoimmune diseases D001327: total 564), infection (parasitic diseases D0101272, Bacterial Infections and mycosis D001423: total 357 molecules), virus diseases (D014777), and cancer (neoplasm D009369: total 3173 molecules). In addition, on basis of the health endpoint framework, the associated biological processes (GO pathways) were selected that are involved in the initiation of the four different immune health endpoint functions (including number of genes). This resulted for hypersensitivity in 164 key processes, for autoimmunity 203 key processes, for infection 187 key processes, and for cancer 309 key processes. This set was used to determine the overlap between the immune disease related genes from the CTD indicated that the set of genes from CTD already incorporated most genes involved in development of immune diseases. As an example of overlapping molecules for the immune health end points hypersensitivity, autoimmunity and infection, a network visualization depicting the unique and shared molecules between these health end points was generated (Figure 2). A selection of candidate biomarkers was generated from the network by filtering down all proteins indicated by the UniProt database as secreted proteins. A network visualization of these secreted proteins with overlap among the different immune health endpoints is displayed in Figure 3. To complete overlap analysis of all immune health endpoints, we visualized the results for clarity reasons in a Venn diagram (Figure 4). The names of the individual genes per immune health endpoints are depicted in the Supplementary Tables 1–4. In total 15 molecules were overlapping between all four immune health endpoints: ALB, HLA-DPB1, HLA-DQA2/ HLA-DQA1, HLA-DRB1, ICAM1, IFNG, IL1B, IL4, IL6, IL10, IL12B, IL18, MPO, CCL2, and TNF.

### Proof of Principle/*in silico* Test Cases

The top interacting genes identified by the CTD for a specific intervention were used for comparison with our previously identified set of genes predicted to be involved in the initiation of immune disorders, to check whether the previously described adverse/beneficial effects confirmed our prediction. Below, several examples of immune modulating substances are given as a proof of principle to illustrate the plausibility that they might interact with any of the other immune health endpoints.

#### Anti-cancer Drugs

Anti-cancer drugs are also called anti-neoplastic agents or chemotherapeutic agents, and act upon rapidly dividing cancer cells to destroy them/for destruction. Examples of anti-cancer drugs are alkylating agents (cisplatin, chlorambucil, procarbazine, carmustine etc.), antimetabolites (methotrexate, cytarabine, gemcitabine etc.), anti-microtubule agents (vinblastine, paclitaxel etc.), topoisomerase inhibitors (etoposide, doxorubicin etc.), cytotoxic agents (bleomycin, mitomycin etc.), and proteasome inhibitors (bortezomib). Anti-cancer drugs have severe adverse effects like hair loss, nausea and vomiting, anemia and increased susceptibility to infection.

**Figure 5:**
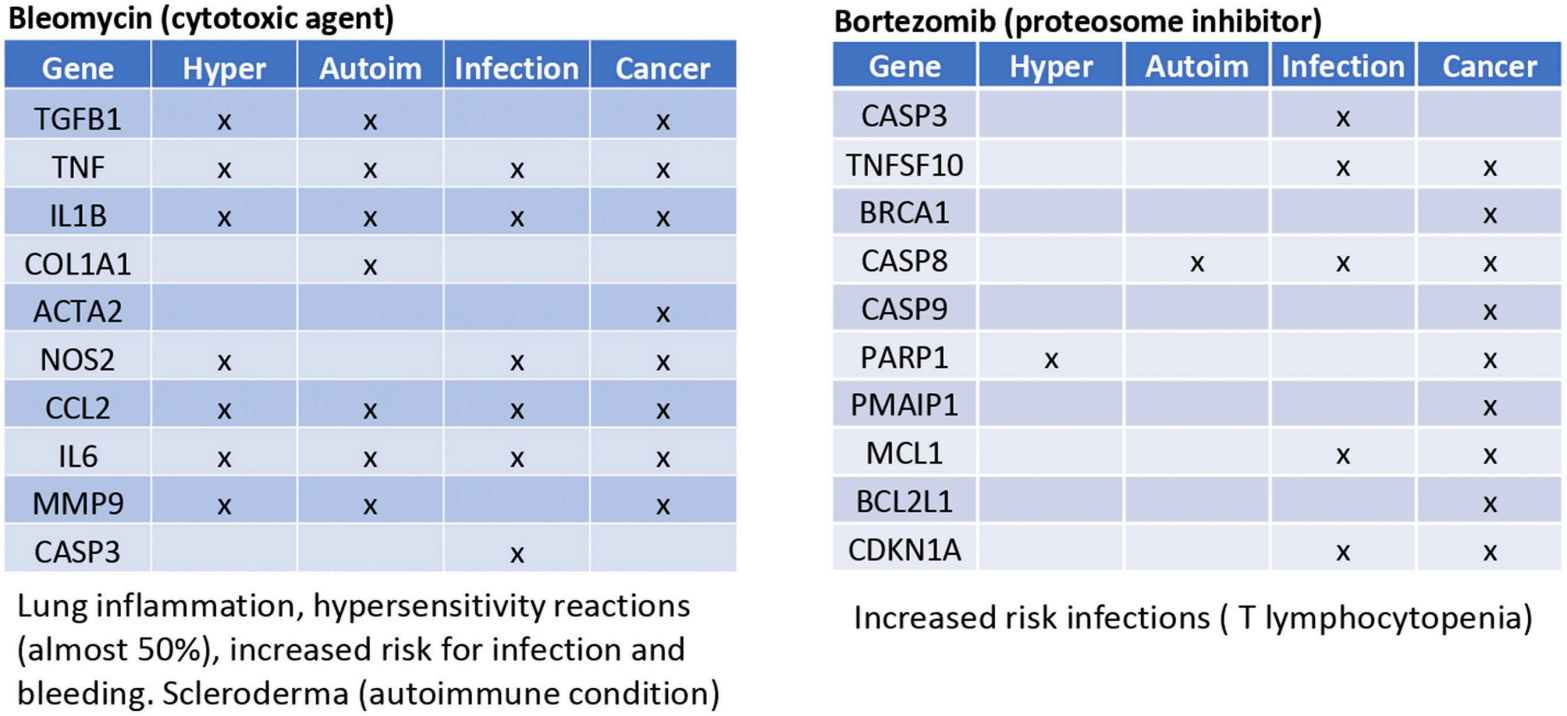
Prediction from CTD anticancer drugs. Gene names are listed of the top 10 interacting genes per chemical. Crosses (×) indicate in which of the four immune health endpoints they are involved.

Bleomicin and bortezomib, as examples of anti-cancer drugs, were checked in the CTD (database) for their top interacting genes. Subsequently, those genes were checked whether they were involved in the onset of the four identified immune health endpoints (Figure 5). As expected, most of the top interacting genes are found in the immune health endpoint infection.

Bleomicin was shown to have interactions with all immune health endpoints. The immune related adverse reactions described for this drug indeed also include hypersensitivity reactions, increased risk for infections and scleroderma (an autoimmune condition). Bortezomib showed to have only interaction with the immune health endpoint infection. The immune related adverse reactions described for bortezomib is an increased risk for infections (T lymphocytopenia).

#### Omega-3 Fatty Acids/PUFA

**Figure 6:**
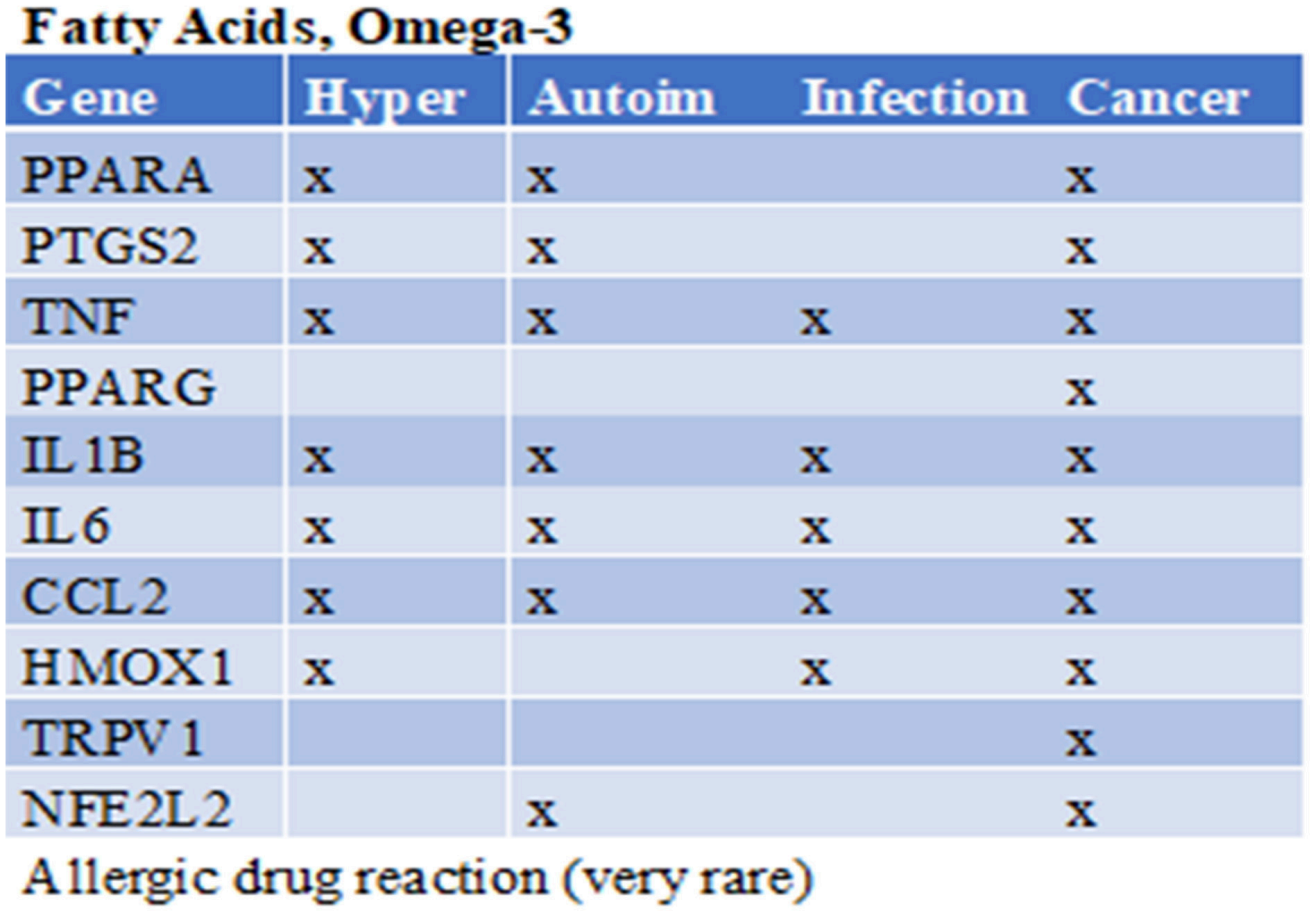
Prediction from CTD omega-3 fatty acids. Gene names are listed of the top 10 interacting genes per chemical. Crosses (X) indicate in which of the four immune health endpoints they are involved. Below the table the immune related side effects observed are listed.

Omega-3 fatty acids are polyunsaturated fatty acids (PUFAs). Omega-3 fatty acids are used as food supplements and have shown some benefits and some health risks (depending on the dietary source) (20). Omega-3 fatty acids were checked in the CTD for their top interacting genes. Subsequently, those genes were checked to see whether they were involved in the immune health endpoints (Figure 6). Interestingly, in all four of the health endpoints the top interacting genes were present. These findings are confirmed in literature: For the immune health point resistance to cancer there is some evidence that the consumption of fish omega-3 fatty acids is associated with a lower risk of breast cancer (21); however, there is insufficient evidence of its benefits on other types of cancer (22). For the immune health point hypersensitivity a clinical trial showed that fish oil consumption during pregnancy reduces persistent wheeze and asthma in the offspring at ages 3–5 years (23). In addition for the immune health point autoimmunity, a systematic review on the intake of omega-3 PUFAs in patients with rheumatoid arthritis, showed that among five proinflammatory markers evaluated, only leukotriene B4 was found to be reduced (24). Last for the immune health point resistance to infection a study showed that omega-3 PUFA-derived lipid mediator protectins could be a new innate suppressor of influenza virus replication that may be further investigated as a potential antiviral drug (25).

These examples illustrate that the prediction with our systems biology approach is promising and can be used to find both negatively as positively correlated interactions. It must be noted that this approach is greatly influenced by the amount of data available on a specific compound; there will be more evidence on the top interacting genes when there are more data available. Therefore, it is of importance that the predicted biomarkers are properly validated in an appropriate assay/study.

### Limitations

The systems biology approach described here is a promising tool, but it does have its limitations. These limitations are based on the fact that it is essential that the data used are accurate, complete, and up to date. Data are continuously generated, so the screening model presented here is a dynamic model and needs continuous on-going scheduled refinements and improvements. Moreover, the approach might be subjected to a reporting bias as it can be difficult to distinguish the absence of a relationship between molecules/pathways from a lack of evaluation of the relationship/pathways. Another limitation is that the data used to generated the toolbox are derived from databases which are mainly disease-driven, and not focused on healthy situations. To overcome this limitation, molecules from the key processes (starting from a healthy condition) driving the development of immune health disturbances were determined and included in the screening/prediction model.

### Future Directions

The approach has been evaluated using a few test cases retrospectively, showing that the side-effects observed in the human population could be retrospectively confirmed by our screening/prediction tool as described in the manuscript. When applied in the context of a testing strategy this screening/prediction tool may be very valuable to identify key molecules/pathways in order to assess safety/beneficial effects of novel immune interventions. For further development of the screening/prediction tool it is important to validate predicted biomarkers in appropriate assays/studies. Moreover, it would be very valuable to incorporate the possibility for stratification of the observations based on parameters known to strongly impact immune functioning, such a age, gender, ethnicity etc.

## Conclusion

We provide a promising systems biology approach to predict genes that help to clarify, on one hand the relationships between immune health interventions and the susceptibility to immune related disorders, and on the other hand, the identification of interesting biomarkers to monitor in safety and efficacy studies on immune interventions.

This systems biology approach forms a good starting point to predict relevant genes and (immune) biomarkers to assess the effects of the immune interventions, which will need to be validated in a follow-up study. Instead of following a tick-box approach for safety assessment, this approach forms the basis of a science-based testing strategy to assess the safety and efficacy of immune interventions. This proposed approach could support a faster way to screen the effects of immune interventions.

## Author Contributions

MM, TvdB, RD, LV, and JvB contributed conception and design of the study. TvdB, RD, and LV organized the database. MM and JvB performed databases searches. MM, LV, and JvB wrote the manuscript. All authors contributed to manuscript revision, read and approved the submitted version.

### Conflict of Interest Statement

LN and AS are employed by Arla Foods Ingredients. JG is employed by Danone Food Safety Center. LK and KK are employed by Danone Nutricia Research. The remaining authors declare that the research was conducted in the absence of any commercial or financial relationships that could be construed as a potential conflict of interest.
