# Supplementary material for: The Impact of Immune Interventions: A Systems Biology Strategy for Predicting Adverse and Beneficial Immune Effects
Source: Front Immunol. 2019 Feb 15;10:231. doi: 10.3389/fimmu.2019.00231 (PMC6384242; doi:10.3389/fimmu.2019.00231)
Supplement: Supplementary file 1 [file Table_1.DOCX]

**Supplementary table I: Genes involved in immune health endpoint Hypersensitivity**

| **EntrezgeneID** | **Gene name** |
| --- | --- |
| 284382 | ACTL9 |
| 108 | ADCY2 |
| 117 | ADCYAP1R1 |
| 154 | ADRB2 |
| 196 | AHR |
| 213 | ALB |
| 217 | ALDH2 |
| 240 | ALOX5 |
| 374 | AREG |
| 383 | ARG1 |
| 384 | ARG2 |
| 374569 | ASPG |
| 596 | BCL2 |
| 632 | BGLAP |
| 716 | C1S |
| 440854 | CAPN14 |
| 84433 | CARD11 |
| 847 | CAT |
| 6356 | CCL11 |
| 6361 | CCL17 |
| 6347 | CCL2 |
| 6367 | CCL22 |
| 6369 | CCL24 |
| 10344 | CCL26 |
| 6352 | CCL5 |
| 6355 | CCL8 |
| 1232 | CCR3 |
| 1234 | CCR5 |
| 929 | CD14 |
| 1015 | CDH17 |
| 1116 | CHI3L1 |
| 1469 | CST1 |
| 29119 | CTNNA3 |
| 2919 | CXCL1 |
| 3627 | CXCL10 |
| 2833 | CXCR3 |
| 10800 | CYSLTR1 |
| 57105 | CYSLTR2 |
| 1767 | DNAH5 |
| 1786 | DNMT1 |
| 1906 | EDN1 |
| 56946 | EMSY |
| 2161 | F12 |
| 2205 | FCER1A |
| 2208 | FCER2 |
| 2312 | FLG |
| 2302 | FOXJ1 |
| 2720 | GLB1 |
| 2944 | GSTM1 |
| 2950 | GSTP1 |
| 8924 | HERC2 |
| 3115 | HLA-DPB1 |
| 3117 | HLA-DQA2/ HLA-DQA1 |
| 3119 | HLA-DQB1 |
| 3123 | HLA-DRB1 |
| 3135 | HLA-G |
| 3162 | HMOX1 |
| 3176 | HNMT |
| 3269 | HRH1 |
| 3291 | HSD11B2 |
| 3309 | HSPA5 |
| 3383 | ICAM1 |
| 3458 | IFNG |
| 22806 | IKZF3 |
| 3586 | IL10 |
| 3593 | IL12B |
| 3596 | IL13 |
| 3600 | IL15 |
| 3606 | IL18 |
| 3553 | IL1B |
| 9173 | IL1RL1 |
| 3557 | IL1RN |
| 50615 | IL21R |
| 90865 | IL33 |
| 3565 | IL4 |
| 3566 | IL4R |
| 3567 | IL5 |
| 3569 | IL6 |
| 11213 | IRAK3 |
| 11127 | KIF3A |
| 3827 | KNG1 |
| 3880 | KRT19 |
| 1241 | LTB4R |
| 4056 | LTC4S |
| 5599 | MAPK8 |
| 4319 | MMP10 |
| 4318 | MMP9 |
| 4353 | MPO |
| 2206 | MS4A2 |
| 4589 | MUC7 |
| 4602 | MYB |
| 114548 | NLRP3 |
| 4843 | NOS2 |
| 1728 | NQO1 |
| 94103 | ORMDL3 |
| 5017 | OVOL1 |
| 142 | PARP1 |
| 5142 | PDE4B |
| 5144 | PDE4D |
| 51131 | PHF11 |
| 7941 | PLA2G7 |
| 5327 | PLAT |
| 5328 | PLAU |
| 5465 | PPARA |
| 5515 | PPP2CA |
| 5728 | PTEN |
| 5729 | PTGDR |
| 11251 | PTGDR2 |
| 5732 | PTGER2 |
| 5739 | PTGIR |
| 5742 | PTGS1 |
| 5743 | PTGS2 |
| 5777 | PTPN6 |
| 149628 | PYHIN1 |
| 10111 | RAD50 |
| 6037 | RNASE3 |
| 6279 | S100A8 |
| 7356 | SCGB1A1 |
| 117156 | SCGB3A2 |
| 6401 | SELE |
| 6403 | SELP |
| 5054 | SERPINE1 |
| 710 | SERPING1 |
| 6556 | SLC11A1 |
| 7782 | SLC30A4 |
| 55630 | SLC39A4 |
| 9021 | SOCS3 |
| 6647 | SOD1 |
| 6648 | SOD2 |
| 11005 | SPINK5 |
| 6701 | SPRR2B |
| 6778 | STAT6 |
| 30009 | TBX21 |
| 6915 | TBXA2R |
| 55714 | TENM3 |
| 7040 | TGFB1 |
| 7078 | TIMP3 |
| 7099 | TLR4 |
| 3371 | TNC |
| 7124 | TNF |
| 7378 | UPP1 |
| 7412 | VCAM1 |
| 7422 | VEGFA |
| 8876 | VNN1 |
| 8875 | VNN2 |
| 134430 | WDR36 |
| 7512 | XPNPEP2 |
| 22891 | ZNF365 |
| 8745 | ADAM23 |
