# Supplementary material for: The Impact of Immune Interventions: A Systems Biology Strategy for Predicting Adverse and Beneficial Immune Effects
Source: Front Immunol. 2019 Feb 15;10:231. doi: 10.3389/fimmu.2019.00231 (PMC6384242; doi:10.3389/fimmu.2019.00231)
Supplement: Supplementary file 2 [file Table_2.DOCX]

**Supplementary table II: Genes involved in immune health endpoint Autoimmunity**

| **EntrezgeneID** | **Gene name** |
| --- | --- |
| 1244 | ABCC2 |
| 10257 | ABCC4 |
| 10057 | ABCC5 |
| 6833 | ABCC8 |
| 9429 | ABCG2 |
| 176 | ACAN |
| 1636 | ACE |
| 57007 | ACKR3 |
| 54 | ACP5 |
| 55 | ACPP |
| 11093 | ADAMTS13 |
| 103 | ADAR |
| 113 | ADCY7 |
| 9370 | ADIPOQ |
| 135 | ADORA2A |
| 3899 | AFF3 |
| 177 | AGER |
| 183 | AGT |
| 196 | AHR |
| 57379 | AICDA |
| 9131 | AIFM1 |
| 326 | AIRE |
| 213 | ALB |
| 229 | ALDOB |
| 239 | ALOX12 |
| 240 | ALOX5 |
| 122416 | ANKRD9 |
| 306 | ANXA3 |
| 335 | APOA1 |
| 200315 | APOBEC3A |
| 348 | APOE |
| 366 | AQP9 |
| 374 | AREG |
| 84159 | ARID5B |
| 471 | ATIC |
| 9212 | AURKB |
| 23080 | AVL9 |
| 567 | B2M |
| 10678 | B3GNT2 |
| 84752 | B3GNT9 |
| 9334 | B4GALT5 |
| 60468 | BACH2 |
| 55971 | BAIAP2L1 |
| 55024 | BANK1 |
| 590 | BCHE |
| 624 | BDKRB2 |
| 633 | BGN |
| 640 | BLK |
| 652 | BMP4 |
| 654 | BMP6 |
| 694 | BTG1 |
| 10950 | BTG3 |
| 29071 | C1GALT1C1 |
| 712 | C1QA |
| 713 | C1QB |
| 716 | C1S |
| 717 | C2 |
| 718 | C3 |
| 720 | C4A |
| 721 | C4B |
| 732 | C8B |
| 84909 | C9ORF3 |
| 203228 | C9ORF72 |
| 800 | CALD1 |
| 157922 | CAMSAP1 |
| 64170 | CARD9 |
| 843 | CASP10 |
| 100506742 | CASP12 |
| 841 | CASP8 |
| 847 | CAT |
| 858 | CAV2 |
| 868 | CBLB |
| 6347 | CCL2 |
| 6364 | CCL20 |
| 6366 | CCL21 |
| 6352 | CCL5 |
| 6355 | CCL8 |
| 1234 | CCR5 |
| 1235 | CCR6 |
| 929 | CD14 |
| 9332 | CD163 |
| 914 | CD2 |
| 10666 | CD226 |
| 100133941 | CD24 |
| 51744 | CD244 |
| 940 | CD28 |
| 916 | CD3E |
| 958 | CD40 |
| 959 | CD40LG |
| 960 | CD44 |
| 921 | CD5 |
| 923 | CD6 |
| 9308 | CD83 |
| 926 | CD8B |
| 1021 | CDK6 |
| 629 | CFB |
| 3078 | CFHR1 |
| 1103 | CHAT |
| 4261 | CIITA |
| 5010 | CLDN11 |
| 23274 | CLEC16A |
| 1191 | CLU |
| 1268 | CNR1 |
| 1277 | COL1A1 |
| 23603 | CORO1C |
| 1356 | CP |
| 1380 | CR2 |
| 1390 | CREM |
| 1401 | CRP |
| 1437 | CSF2 |
| 1445 | CSK |
| 1490 | CTGF |
| 1493 | CTLA4 |
| 1509 | CTSD |
| 1512 | CTSH |
| 2017 | CTTN |
| 2919 | CXCL1 |
| 2920 | CXCL2 |
| 6372 | CXCL6 |
| 3576 | CXCL8 |
| 3579 | CXCR2 |
| 2833 | CXCR3 |
| 7852 | CXCR4 |
| 3491 | CYR61 |
| 1612 | DAPK1 |
| 1649 | DDIT3 |
| 54541 | DDIT4 |
| 1656 | DDX6 |
| 50619 | DEF6 |
| 1670 | DEFA5 |
| 1687 | GSDME |
| 1609 | DGKQ |
| 1719 | DHFR |
| 1773 | DNASE1 |
| 1776 | DNASE1L3 |
| 1843 | DUSP1 |
| 1844 | DUSP2 |
| 1846 | DUSP4 |
| 8291 | DYSF |
| 1950 | EGF |
| 1958 | EGR1 |
| 9451 | EIF2AK3 |
| 1998 | ELF2 |
| 2023 | ENO1 |
| 2056 | EPO |
| 55500 | ETNK1 |
| 2113 | ETS1 |
| 2120 | ETV6 |
| 2147 | F2 |
| 2157 | F8 |
| 54757 | FAM20A |
| 355 | FAS |
| 356 | FASLG |
| 10826 | FAXDC2 |
| 2091 | FBL |
| 2209 | FCGR1A |
| 2212 | FCGR2A |
| 2213 | FCGR2B |
| 9103 | FCGR2A |
| 2214 | FCGR3A |
| 2215 | FCGR3B |
| 9873 | FCHSD2 |
| 115352 | FCRL3 |
| 26291 | FGF21 |
| 2289 | FKBP5 |
| 2353 | FOS |
| 2354 | FOSB |
| 2355 | FOSL2 |
| 27022 | FOXD3 |
| 2305 | FOXM1 |
| 27086 | FOXP1 |
| 50943 | FOXP3 |
| 2356 | FPGS |
| 2548 | GAA |
| 1647 | GADD45A |
| 2618 | GART |
| 2625 | GATA3 |
| 2638 | GC |
| 8200 | GDF5 |
| 2670 | GFAP |
| 8836 | GGH |
| 54826 | GIN1 |
| 169792 | GLIS3 |
| 2766 | GMPR |
| 2812 | GP1BB |
| 1880 | GPR183 |
| 9052 | GPRC5A |
| 156 | GRK2 |
| 2870 | GRK6 |
| 2969 | GTF2I |
| 3035 | HARS |
| 1839 | HBEGF |
| 3059 | HCLS1 |
| 3012 | HIST1H2AE |
| 8339 | HIST1H2BG |
| 3106 | HLA-B |
| 3115 | HLA-DPB1 |
| 3117 | HLA-DQA1 |
| 3118 | HLA-DQA2 |
| 3119 | HLA-DQB1 |
| 3122 | HLA-DRA |
| 3123 | HLA-DRB1 |
| 3156 | HMGCR |
| 6927 | HNF1A |
| 3236 | HOXD10 |
| 3237 | HOXD11 |
| 3239 | HOXD13 |
| 3240 | HP |
| 10855 | HPSE |
| 3263 | HPX |
| 3290 | HSD11B1 |
| 3291 | HSD11B2 |
| 3310 | HSPA6 |
| 3313 | HSPA9 |
| 3383 | ICAM1 |
| 64135 | IFIH1 |
| 3440 | IFNA2 |
| 3456 | IFNB1 |
| 3458 | IFNG |
| 3479 | IGF1 |
| 3480 | IGF1R |
| 3486 | IGFBP3 |
| 10320 | IKZF1 |
| 22806 | IKZF3 |
| 3586 | IL10 |
| 3592 | IL12A |
| 3593 | IL12B |
| 3596 | IL13 |
| 3605 | IL17A |
| 3606 | IL18 |
| 29949 | IL19 |
| 3553 | IL1B |
| 3557 | IL1RN |
| 3558 | IL2 |
| 50604 | IL20 |
| 59067 | IL21 |
| 50615 | IL21R |
| 50616 | IL22 |
| 51561 | IL23A |
| 246778 | IL27 |
| 3559 | IL2RA |
| 3560 | IL2RB |
| 3565 | IL4 |
| 3567 | IL5 |
| 3569 | IL6 |
| 3570 | IL6R |
| 3572 | IL6ST |
| 3574 | IL7 |
| 3575 | IL7R |
| 3578 | IL9 |
| 3630 | INS |
| 3654 | IRAK1 |
| 134728 | IRAK1BP1 |
| 3663 | IRF5 |
| 3394 | IRF8 |
| 83737 | ITCH |
| 3674 | ITGA2B |
| 3655 | ITGA6 |
| 3684 | ITGAM |
| 3694 | ITGB6 |
| 50618 | ITSN2 |
| 221895 | JAZF1 |
| 221037 | JMJD1C |
| 3725 | JUN |
| 23522 | KAT6B |
| 3766 | KCNJ10 |
| 3767 | KCNJ11 |
| 56992 | KIF15 |
| 23095 | KIF1B |
| 3798 | KIF5A |
| 9314 | KLF4 |
| 3845 | KRAS |
| 3880 | KRT19 |
| 3934 | LCN2 |
| 3952 | LEP |
| 10184 | LHFPL2 |
| 9355 | LHX2 |
| 23643 | LY96 |
| 10586 | MAB21L2 |
| 8379 | MAD1L1 |
| 23764 | MAFF |
| 84441 | MAML2 |
| 4125 | MAN2B1 |
| 4133 | MAP2 |
| 5594 | MAPK1 |
| 4082 | MARCKS |
| 4155 | MBP |
| 10461 | MERTK |
| 4282 | MIF |
| 79258 | MMEL1 |
| 4319 | MMP10 |
| 4321 | MMP12 |
| 4313 | MMP2 |
| 4318 | MMP9 |
| 4353 | MPO |
| 4478 | MSN |
| 4524 | MTHFR |
| 9788 | MTSS1 |
| 4593 | MUSK |
| 100820829 | MYZAP |
| 653361 | NCF1 |
| 4688 | NCF2 |
| 81565 | NDEL1 |
| 56901 | NDUFA4L2 |
| 4780 | NFE2L2 |
| 4795 | NFKBIL1 |
| 22861 | NLRP1 |
| 4842 | NOS1 |
| 4846 | NOS3 |
| 4929 | NR4A2 |
| 8013 | NR4A3 |
| 4893 | NRAS |
| 3084 | NRG1 |
| 4938 | OAS1 |
| 100506658 | OCLN |
| 4973 | OLR1 |
| 5008 | OSM |
| 5027 | P2RX7 |
| 64805 | P2RY12 |
| 11252 | PACSIN2 |
| 23569 | PADI4 |
| 5078 | PAX4 |
| 9468 | PCYT1B |
| 5133 | PDCD1 |
| 5175 | PECAM1 |
| 5187 | PER1 |
| 5196 | PF4 |
| 5239 | PGM5 |
| 55023 | PHIP |
| 57661 | PHRF1 |
| 5284 | PIGR |
| 5328 | PLAU |
| 5329 | PLAUR |
| 151056 | PLB1 |
| 122618 | PLD4 |
| 5341 | PLEK |
| 10769 | PLK2 |
| 5352 | PLOD2 |
| 5428 | POLG |
| 5443 | POMC |
| 5444 | PON1 |
| 5453 | POU3F1 |
| 5465 | PPARA |
| 9055 | PRC1 |
| 639 | PRDM1 |
| 5588 | PRKCQ |
| 5617 | PRL |
| 5627 | PROS1 |
| 5657 | PRTN3 |
| 5743 | PTGS2 |
| 5747 | PTK2 |
| 5771 | PTPN2 |
| 26191 | PTPN22 |
| 5788 | PTPRC |
| 9232 | PTTG1 |
| 54899 | PXK |
| 4218 | RAB8A |
| 5896 | RAG1 |
| 5903 | RANBP2 |
| 5911 | RAP2A |
| 5913 | RAPSN |
| 153020 | RASGEF1B |
| 10125 | RASGRP1 |
| 25780 | RASGRP3 |
| 54502 | RBM47 |
| 5949 | RBP3 |
| 3516 | RBPJ |
| 1827 | RCAN1 |
| 5966 | REL |
| 473 | RERE |
| 285704 | RGMB |
| 10535 | RNASEH2A |
| 79621 | RNASEH2B |
| 84153 | RNASEH2C |
| 8635 | RNASET2 |
| 7844 | RNF103 |
| 861 | RUNX1 |
| 860 | RUNX2 |
| 25939 | SAMHD1 |
| 6319 | SCD |
| 6401 | SELE |
| 710 | SERPING1 |
| 10019 | SH2B3 |
| 54414 | SIAE |
| 150094 | SIK1 |
| 26037 | SIPA1L1 |
| 23411 | SIRT1 |
| 6556 | SLC11A1 |
| 6583 | SLC22A4 |
| 8604 | SLC25A12 |
| 55315 | SLC29A3 |
| 6515 | SLC2A3 |
| 4088 | SMAD3 |
| 6611 | SMS |
| 6648 | SOD2 |
| 200734 | SPRED2 |
| 10610 | ST6GALNAC2 |
| 81849 | ST6GALNAC5 |
| 23166 | STAB1 |
| 6772 | STAT1 |
| 6774 | STAT3 |
| 6775 | STAT4 |
| 6786 | STIM1 |
| 412 | STS |
| 387082 | SUMO4 |
| 6932 | TCF7 |
| 6934 | TCF7L2 |
| 7015 | TERT |
| 7027 | TFDP1 |
| 7035 | TFPI |
| 7980 | TFPI2 |
| 7038 | TG |
| 7040 | TGFB1 |
| 7042 | TGFB2 |
| 7057 | THBS1 |
| 7076 | TIMP1 |
| 7090 | TLE3 |
| 7097 | TLR2 |
| 7100 | TLR5 |
| 7112 | TMPO |
| 7124 | TNF |
| 7128 | TNFAIP3 |
| 7130 | TNFAIP6 |
| 25816 | TNFAIP8 |
| 8794 | TNFRSF10C |
| 4982 | TNFRSF11B |
| 8764 | TNFRSF14 |
| 7132 | TNFRSF1A |
| 7293 | TNFRSF4 |
| 8741 | TNFSF13 |
| 8740 | TNFSF14 |
| 7292 | TNFSF4 |
| 23043 | TNIK |
| 10318 | TNIP1 |
| 7185 | TRAF1 |
| 7189 | TRAF6 |
| 11277 | TREX1 |
| 7253 | TSHR |
| 23093 | TTLL5 |
| 7280 | TUBB2A |
| 81567 | TXNDC5 |
| 10628 | TXNIP |
| 7297 | TYK2 |
| 7324 | UBE2E1 |
| 9690 | UBE3C |
| 10451 | VAV3 |
| 7412 | VCAM1 |
| 7422 | VEGFA |
| 11326 | VSIG4 |
| 10810 | WASF3 |
| 51741 | WWOX |
| 57623 | ZFAT |
| 196441 | ZFC3H1 |
