# Supplementary material for: The Impact of Immune Interventions: A Systems Biology Strategy for Predicting Adverse and Beneficial Immune Effects
Source: Front Immunol. 2019 Feb 15;10:231. doi: 10.3389/fimmu.2019.00231 (PMC6384242; doi:10.3389/fimmu.2019.00231)
Supplement: Supplementary file 3 [file Table_3.DOCX]

**Supplementary table III: Genes involved in immune health endpoint resistance to Infection**

| **EntrezgeneID** | **Gene name** |
| --- | --- |
| 1636 | ACE |
| 8639 | AOC3 |
| 50807 | ASAP1 |
| 55509 | BATF3 |
| 715 | C1R |
| 718 | C3 |
| 731 | C8A |
| 732 | C8B |
| 735 | C9 |
| 836 | CASP3 |
| 283234 | CCDC88B |
| 6347 | CCL2 |
| 30835 | CD209 |
| 948 | CD36 |
| 999 | CDH1 |
| 1027 | CDKN1B |
| 1675 | CFD |
| 3075 | CFH |
| 5199 | CFP |
| 1154 | CISH |
| 11151 | CORO1A |
| 1356 | CP |
| 1401 | CRP |
| 1522 | CTSZ |
| 3576 | CXCL8 |
| 1540 | CYLD |
| 51164 | DCTN4 |
| 1673 | DEFB4A |
| 1718 | DHCR24 |
| 2147 | F2 |
| 10211 | FLOT1 |
| 2706 | GJB2 |
| 2934 | GSN |
| 3117 | HLA-DQA1 |
| 3123 | HLA-DRB1 |
| 3156 | HMGCR |
| 3240 | HP |
| 3458 | IFNG |
| 8517 | IKBKG |
| 3593 | IL12B |
| 3594 | IL12RB1 |
| 149233 | IL23R |
| 3565 | IL4 |
| 51135 | IRAK4 |
| 345611 | IRGM |
| 3949 | LDLR |
| 4047 | LSS |
| 4049 | LTA |
| 5594 | MAPK1 |
| 5595 | MAPK3 |
| 10747 | MASP2 |
| 4159 | MC3R |
| 23385 | NCSTN |
| 4843 | NOS2 |
| 4864 | NPC1 |
| 5071 | PRKN |
| 5327 | PLAT |
| 5663 | PSEN1 |
| 55851 | PSENEN |
| 5803 | PTPRZ1 |
| 10981 | RAB32 |
| 10411 | RAPGEF3 |
| 6037 | RNASE3 |
| 23411 | SIRT1 |
| 6556 | SLC11A1 |
| 3431 | SP110 |
| 6772 | STAT1 |
| 6905 | TBCE |
| 7018 | TF |
| 7037 | TFRC |
| 114609 | TIRAP |
| 7096 | TLR1 |
| 7097 | TLR2 |
| 7100 | TLR5 |
| 7124 | TNF |
| 2532 | ACKR1 |
| 213 | ALB |
| 383 | ARG1 |
| 596 | BCL2 |
| 920 | CD4 |
| 1378 | CR1 |
| 1437 | CSF2 |
| 1548 | CYP2A6 |
| 355 | FAS |
| 2213 | FCGR2B |
| 2778 | GNAS |
| 2995 | GYPC |
| 3043 | HBB |
| 3162 | HMOX1 |
| 3383 | ICAM1 |
| 3586 | IL10 |
| 3606 | IL18 |
| 3553 | IL1B |
| 3558 | IL2 |
| 3567 | IL5 |
| 3569 | IL6 |
| 58508 | KMT2C |
| 3845 | KRAS |
| 4055 | LTBR |
| 4170 | MCL1 |
| 4313 | MMP2 |
| 4353 | MPO |
| 259197 | NCR3 |
| 5178 | PEG3 |
| 5052 | PRDX1 |
| 11186 | RASSF1 |
| 54894 | RNF43 |
| 6521 | SLC4A1 |
| 4089 | SMAD4 |
| 6647 | SOD1 |
| 7078 | TIMP3 |
| 54106 | TLR9 |
| 8784 | TNFRSF18 |
| 7157 | TP53 |
| 53947 | A4GALT |
| 10189 | ALYREF |
| 335 | APOA1 |
| 80830 | APOL6 |
| 64411 | ARAP3 |
| 393 | ARHGAP4 |
| 396 | ARHGDIA |
| 397 | ARHGDIB |
| 8289 | ARID1A |
| 467 | ATF3 |
| 9531 | BAG3 |
| 581 | BAX |
| 329 | BIRC2 |
| 330 | BIRC3 |
| 841 | CASP8 |
| 6356 | CCL11 |
| 6348 | CCL3 |
| 6349 | CCL3L1 |
| 6351 | CCL4 |
| 6355 | CCL8 |
| 8900 | CCNA1 |
| 1232 | CCR3 |
| 1234 | CCR5 |
| 7203 | CCT3 |
| 22948 | CCT5 |
| 10574 | CCT7 |
| 940 | CD28 |
| 4179 | CD46 |
| 975 | CD81 |
| 998 | CDC42 |
| 1026 | CDKN1A |
| 23601 | CLEC5A |
| 10980 | COPS6 |
| 1490 | CTGF |
| 1524 | CX3CR1 |
| 3627 | CXCL10 |
| 6387 | CXCL12 |
| 3577 | CXCR1 |
| 7852 | CXCR4 |
| 1576 | CYP3A4 |
| 1605 | DAG1 |
| 23586 | DDX58 |
| 55601 | DDX60 |
| 7266 | DNAJC7 |
| 10919 | EHMT2 |
| 5610 | EIF2AK2 |
| 8662 | EIF3B |
| 1973 | EIF4A1 |
| 1978 | EIF4EBP1 |
| 3692 | EIF6 |
| 10436 | EMG1 |
| 2208 | FCER2 |
| 10841 | FTCD |
| 2524 | FUT2 |
| 1647 | GADD45A |
| 2633 | GBP1 |
| 2971 | GTF3A |
| 3068 | HDGF |
| 51191 | HERC5 |
| 55008 | HERC6 |
| 3077 | HFE |
| 3082 | HGF |
| 3106 | HLA-B |
| 3107 | HLA-C |
| 3113 | HLA-DPA1 |
| 3115 | HLA-DPB1 |
| 3320 | HSP90AA1 |
| 3326 | HSP90AB1 |
| 3313 | HSPA9 |
| 3315 | HSPB1 |
| 3329 | HSPD1 |
| 10808 | HSPH1 |
| 3399 | ID3 |
| 3429 | IFI27 |
| 3430 | IFI35 |
| 2537 | IFI6 |
| 64135 | IFIH1 |
| 3434 | IFIT1 |
| 3433 | IFIT2 |
| 3437 | IFIT3 |
| 24138 | IFIT5 |
| 10410 | IFITM3 |
| 3439 | IFNA1 |
| 3440 | IFNA2 |
| 3454 | IFNAR1 |
| 3455 | IFNAR2 |
| 3456 | IFNB1 |
| 3588 | IL10RB |
| 3600 | IL15 |
| 3552 | IL1A |
| 3559 | IL2RA |
| 3566 | IL4R |
| 3608 | ILF2 |
| 3665 | IRF7 |
| 9636 | ISG15 |
| 3694 | ITGB6 |
| 3811 | KIR3DL1 |
| 3837 | KPNB1 |
| 27074 | LAMP3 |
| 4000 | LMNA |
| 4015 | LOX |
| 4017 | LOXL2 |
| 4023 | LPL |
| 4061 | LY6E |
| 4067 | LYN |
| 4150 | MAZ |
| 4153 | MBL2 |
| 4193 | MDM2 |
| 4277 | MICB |
| 4599 | MX1 |
| 4600 | MX2 |
| 4609 | MYC |
| 5818 | NECTIN1 |
| 4927 | NUP88 |
| 4938 | OAS1 |
| 4939 | OAS2 |
| 4940 | OAS3 |
| 8638 | OASL |
| 4953 | ODC1 |
| 4988 | OPRM1 |
| 5087 | PBX1 |
| 5091 | PC |
| 201626 | PDE12 |
| 5295 | PIK3R1 |
| 51196 | PLCE1 |
| 5338 | PLD2 |
| 5359 | PLSCR1 |
| 5479 | PPIB |
| 10594 | PRPF8 |
| 5685 | PSMA4 |
| 5686 | PSMA5 |
| 5687 | PSMA6 |
| 5699 | PSMB10 |
| 5692 | PSMB4 |
| 5702 | PSMC3 |
| 5705 | PSMC5 |
| 5719 | PSMD13 |
| 5709 | PSMD3 |
| 9861 | PSMD6 |
| 5714 | PSMD8 |
| 5720 | PSME1 |
| 5721 | PSME2 |
| 10197 | PSME3 |
| 23198 | PSME4 |
| 5727 | PTCH1 |
| 5788 | PTPRC |
| 5817 | PVR |
| 9230 | RAB11B |
| 5896 | RAG1 |
| 5901 | RAN |
| 5902 | RANBP1 |
| 5979 | RET |
| 91543 | RSAD2 |
| 27164 | SALL3 |
| 54809 | SAMD9 |
| 23098 | SARM1 |
| 949 | SCARB1 |
| 6335 | SCN9A |
| 9997 | SCO2 |
| 5265 | SERPINA1 |
| 710 | SERPING1 |
| 10946 | SF3A3 |
| 6421 | SFPQ |
| 6614 | SIGLEC1 |
| 6581 | SLC22A3 |
| 6583 | SLC22A4 |
| 6584 | SLC22A5 |
| 6597 | SMARCA4 |
| 6634 | SNRPD3 |
| 6722 | SRF |
| 6426 | SRSF1 |
| 8683 | SRSF9 |
| 6880 | TAF9 |
| 6929 | TCF3 |
| 23424 | TDRD7 |
| 148022 | TICAM1 |
| 7098 | TLR3 |
| 7130 | TNFAIP6 |
| 8743 | TNFSF10 |
| 54209 | TREM2 |
| 11277 | TREX1 |
| 10346 | TRIM22 |
| 85363 | TRIM5 |
| 7305 | TYROBP |
| 7307 | U2AF1 |
| 7332 | UBE2L3 |
| 9246 | UBE2L6 |
| 81622 | UNC93B1 |
| 6845 | VAMP7 |
| 54739 | XAF1 |
| 7528 | YY1 |
| 10269 | ZMPSTE24 |
