# Supplementary material for: The Impact of Immune Interventions: A Systems Biology Strategy for Predicting Adverse and Beneficial Immune Effects
Source: Front Immunol. 2019 Feb 15;10:231. doi: 10.3389/fimmu.2019.00231 (PMC6384242; doi:10.3389/fimmu.2019.00231)
Supplement: Supplementary file 4 [file Table_4.DOCX]

**Supplementary table VI: Genes involved in immune health endpoint resistance to Cancer**

| **EntrezgeneID** | **Gene name** |
| --- | --- |
| 2 | A2M |
| 53947 | A4GALT |
| 8086 | AAAS |
| 19 | ABCA1 |
| 21 | ABCA3 |
| 24 | ABCA4 |
| 23461 | ABCA5 |
| 8647 | ABCB11 |
| 5244 | ABCB4 |
| 10058 | ABCB6 |
| 4363 | ABCC1 |
| 1244 | ABCC2 |
| 10257 | ABCC4 |
| 10057 | ABCC5 |
| 6833 | ABCC8 |
| 225 | ABCD2 |
| 5825 | ABCD3 |
| 5826 | ABCD4 |
| 6059 | ABCE1 |
| 9619 | ABCG1 |
| 9429 | ABCG2 |
| 25 | ABL1 |
| 28 | ABO |
| 29 | ABR |
| 29777 | ABT1 |
| 31 | ACACA |
| 27034 | ACAD8 |
| 1636 | ACE |
| 55331 | ACER3 |
| 43 | ACHE |
| 57007 | ACKR3 |
| 47 | ACLY |
| 48 | ACO1 |
| 50 | ACO2 |
| 641371 | ACOT1 |
| 51 | ACOX1 |
| 55289 | ACOXL |
| 2180 | ACSL1 |
| 2182 | ACSL4 |
| 116285 | ACSM1 |
| 6296 | ACSM3 |
| 59 | ACTA2 |
| 60 | ACTB |
| 70 | ACTC1 |
| 71 | ACTG1 |
| 72 | ACTG2 |
| 81 | ACTN4 |
| 90 | ACVR1 |
| 95 | ACY1 |
| 100 | ADA |
| 102 | ADAM10 |
| 10863 | ADAM28 |
| 8754 | ADAM9 |
| 9510 | ADAMTS1 |
| 170691 | ADAMTS17 |
| 9509 | ADAMTS2 |
| 80070 | ADAMTS20 |
| 11173 | ADAMTS7 |
| 104 | ADARB1 |
| 107 | ADCY1 |
| 111 | ADCY5 |
| 113 | ADCY7 |
| 116 | ADCYAP1 |
| 118 | ADD1 |
| 125 | ADH1B |
| 126 | ADH1C |
| 131 | ADH7 |
| 55256 | ADI1 |
| 136 | ADORA2B |
| 153 | ADRB1 |
| 154 | ADRB2 |
| 174 | AFP |
| 26523 | AGO1 |
| 27161 | AGO2 |
| 10551 | AGR2 |
| 375790 | AGRN |
| 185 | AGTR1 |
| 186 | AGTR2 |
| 23382 | AHCYL2 |
| 79026 | AHNAK |
| 196 | AHR |
| 57379 | AICDA |
| 9049 | AIP |
| 326 | AIRE |
| 84962 | AJUBA |
| 203 | AK1 |
| 9590 | AKAP12 |
| 11214 | AKAP13 |
| 231 | AKR1B1 |
| 57016 | AKR1B10 |
| 1645 | AKR1C1 |
| 1646 | AKR1C2 |
| 8644 | AKR1C3 |
| 207 | AKT1 |
| 208 | AKT2 |
| 210 | ALAD |
| 212 | ALAS2 |
| 213 | ALB |
| 216 | ALDH1A1 |
| 8854 | ALDH1A2 |
| 220 | ALDH1A3 |
| 219 | ALDH1B1 |
| 217 | ALDH2 |
| 8659 | ALDH4A1 |
| 501 | ALDH7A1 |
| 226 | AC093512.2 |
| 229 | ALDOB |
| 238 | ALK |
| 246 | ALOX15 |
| 240 | ALOX5 |
| 59344 | ALOXE3 |
| 60529 | ALX4 |
| 23600 | AMACR |
| 259 | AMBP |
| 267 | AMFR |
| 283 | ANG |
| 51129 | ANGPTL4 |
| 51479 | ANKFY1 |
| 54443 | ANLN |
| 290 | ANPEP |
| 84168 | ANTXR1 |
| 118429 | ANTXR2 |
| 301 | ANXA1 |
| 302 | ANXA2 |
| 306 | ANXA3 |
| 307 | ANXA4 |
| 309 | ANXA6 |
| 310 | ANXA7 |
| 316 | AOX1 |
| 55745 | AP5M1 |
| 317 | APAF1 |
| 324 | APC |
| 10297 | APC2 |
| 325 | APCS |
| 328 | APEX1 |
| 335 | APOA1 |
| 337 | APOA4 |
| 200315 | APOBEC3A |
| 344 | APOC2 |
| 345 | APOC3 |
| 347 | APOD |
| 348 | APOE |
| 55198 | APPL2 |
| 353 | APRT |
| 358 | AQP1 |
| 360 | AQP3 |
| 366 | AQP9 |
| 367 | AR |
| 369 | ARAF |
| 374 | AREG |
| 383 | ARG1 |
| 384 | ARG2 |
| 23092 | ARHGAP26 |
| 2909 | ARHGAP35 |
| 396 | ARHGDIA |
| 7984 | ARHGEF5 |
| 8289 | ARID1A |
| 57492 | ARID1B |
| 196528 | ARID2 |
| 51742 | ARID4B |
| 84159 | ARID5B |
| 402 | ARL2 |
| 23204 | ARL6IP1 |
| 10550 | ARL6IP5 |
| 405 | ARNT |
| 406 | ARNTL |
| 57561 | ARRDC3 |
| 9048 | ARTN |
| 57412 | AS3MT |
| 50807 | ASAP1 |
| 142685 | ASB15 |
| 429 | ASCL1 |
| 430 | ASCL2 |
| 25842 | ASF1A |
| 55870 | ASH1L |
| 434 | ASIP |
| 8623 | ASMTL |
| 259266 | ASPM |
| 79058 | ASPSCR1 |
| 171023 | ASXL1 |
| 55252 | ASXL2 |
| 80816 | ASXL3 |
| 136991 | ASZ1 |
| 79915 | ATAD5 |
| 466 | ATF1 |
| 1386 | ATF2 |
| 467 | ATF3 |
| 468 | ATF4 |
| 55729 | ATF7IP |
| 55102 | ATG2B |
| 472 | ATM |
| 474 | ATOH1 |
| 476 | ATP1A1 |
| 481 | ATP1B1 |
| 506 | ATP5F1B |
| 513 | ATP5F1D |
| 10476 | ATP5PD |
| 537 | ATP6AP1 |
| 245972 | ATP6V0D2 |
| 526 | ATP6V1B2 |
| 538 | ATP7A |
| 540 | ATP7B |
| 93974 | ATP5IF1 |
| 545 | ATR |
| 546 | ATRX |
| 6790 | AURKA |
| 9212 | AURKB |
| 551 | AVP |
| 8312 | AXIN1 |
| 8313 | AXIN2 |
| 558 | AXL |
| 563 | AZGP1 |
| 567 | B2M |
| 8702 | B4GALT4 |
| 29086 | BABAM1 |
| 60468 | BACH2 |
| 572 | BAD |
| 578 | BAK1 |
| 8314 | BAP1 |
| 580 | BARD1 |
| 56033 | BARX1 |
| 10409 | BASP1 |
| 116071 | BATF2 |
| 581 | BAX |
| 11176 | BAZ2A |
| 80127 | BBOF1 |
| 9564 | BCAR1 |
| 8412 | BCAR3 |
| 587 | BCAT2 |
| 590 | BCHE |
| 53335 | BCL11A |
| 64919 | BCL11B |
| 596 | BCL2 |
| 598 | BCL2L1 |
| 604 | BCL6 |
| 607 | BCL9 |
| 9774 | BCLAF1 |
| 54880 | BCOR |
| 63035 | BCORL1 |
| 613 | BCR |
| 8678 | BECN1 |
| 632 | BGLAP |
| 8553 | BHLHE40 |
| 635 | BHMT |
| 637 | BID |
| 329 | BIRC2 |
| 330 | BIRC3 |
| 332 | BIRC5 |
| 57448 | BIRC6 |
| 79444 | BIRC7 |
| 388552 | BLOC1S3 |
| 645 | BLVRB |
| 90427 | BMF |
| 648 | BMI1 |
| 9210 | BMP15 |
| 650 | BMP2 |
| 652 | BMP4 |
| 654 | BMP6 |
| 655 | BMP7 |
| 657 | BMPR1A |
| 658 | BMPR1B |
| 659 | BMPR2 |
| 54796 | BNC2 |
| 664 | BNIP3 |
| 66037 | BOLL |
| 23246 | BOP1 |
| 2186 | BPTF |
| 673 | BRAF |
| 672 | BRCA1 |
| 675 | BRCA2 |
| 23774 | BRD1 |
| 6046 | BRD2 |
| 8019 | BRD3 |
| 23476 | BRD4 |
| 65980 | BRD9 |
| 83990 | BRIP1 |
| 682 | BSG |
| 121551 | BTBD11 |
| 694 | BTG1 |
| 7832 | BTG2 |
| 10950 | BTG3 |
| 701 | BUB1B |
| 9689 | BZW1 |
| 83636 | C19ORF12 |
| 374920 | ZSWIM9 |
| 713 | C1QB |
| 708 | C1QBP |
| 735 | C9 |
| 84909 | C9ORF3 |
| 759 | CA1 |
| 760 | CA2 |
| 768 | CA9 |
| 26256 | CABYR |
| 781 | CACNA2D1 |
| 790 | CAD |
| 23705 | CADM1 |
| 794 | CALB2 |
| 796 | CALCA |
| 811 | CALR |
| 813 | CALU |
| 817 | CAMK2D |
| 822 | CAPG |
| 823 | CAPN1 |
| 824 | CAPN2 |
| 826 | CAPNS1 |
| 829 | CAPZA1 |
| 832 | CAPZB |
| 84433 | CARD11 |
| 59082 | CARD18 |
| 8573 | CASK |
| 843 | CASP10 |
| 835 | CASP2 |
| 840 | CASP7 |
| 841 | CASP8 |
| 842 | CASP9 |
| 847 | CAT |
| 857 | CAV1 |
| 858 | CAV2 |
| 865 | CBFB |
| 867 | CBL |
| 873 | CBR1 |
| 875 | CBS |
| 10951 | CBX1 |
| 23492 | CBX7 |
| 8030 | CCDC6 |
| 160762 | CCDC63 |
| 54535 | CCHCR1 |
| 6362 | CCL18 |
| 6347 | CCL2 |
| 6364 | CCL20 |
| 6368 | CCL23 |
| 6348 | CCL3 |
| 6352 | CCL5 |
| 6354 | CCL7 |
| 83605 | CCM2 |
| 8900 | CCNA1 |
| 891 | CCNB1 |
| 85417 | CCNB3 |
| 595 | CCND1 |
| 894 | CCND2 |
| 898 | CCNE1 |
| 9134 | CCNE2 |
| 900 | CCNG1 |
| 902 | CCNH |
| 1230 | CCR1 |
| 1233 | CCR4 |
| 1236 | CCR7 |
| 22948 | CCT5 |
| 10574 | CCT7 |
| 135228 | CD109 |
| 283316 | CD163L1 |
| 10666 | CD226 |
| 29126 | CD274 |
| 80381 | CD276 |
| 940 | CD28 |
| 945 | CD33 |
| 947 | CD34 |
| 958 | CD40 |
| 960 | CD44 |
| 921 | CD5 |
| 967 | CD63 |
| 972 | CD74 |
| 973 | CD79A |
| 974 | CD79B |
| 942 | CD86 |
| 928 | CD9 |
| 978 | CDA |
| 998 | CDC42 |
| 990 | CDC6 |
| 79577 | CDC73 |
| 999 | CDH1 |
| 1012 | CDH13 |
| 1000 | CDH2 |
| 1001 | CDH3 |
| 1003 | CDH5 |
| 983 | CDK1 |
| 5218 | CDK14 |
| 1017 | CDK2 |
| 10263 | CDK2AP2 |
| 1019 | CDK4 |
| 1021 | CDK6 |
| 8999 | CDKL2 |
| 1026 | CDKN1A |
| 1027 | CDKN1B |
| 1028 | CDKN1C |
| 1029 | CDKN2A |
| 1030 | CDKN2B |
| 1032 | CDKN2D |
| 1040 | CDS1 |
| 9425 | CDYL |
| 634 | CEACAM1 |
| 1050 | CEBPA |
| 1051 | CEBPB |
| 1052 | CEBPD |
| 1053 | CEBPE |
| 1063 | CENPF |
| 55165 | CEP55 |
| 1066 | CES1 |
| 1675 | CFD |
| 1072 | CFL1 |
| 8837 | CFLAR |
| 1080 | CFTR |
| 1081 | CGA |
| 1107 | CHD3 |
| 1108 | CHD4 |
| 26038 | CHD5 |
| 84181 | CHD6 |
| 1111 | CHEK1 |
| 11200 | CHEK2 |
| 55743 | CHFR |
| 1113 | CHGA |
| 1116 | CHI3L1 |
| 92421 | CHMP4C |
| 51510 | CHMP5 |
| 1136 | CHRNA3 |
| 1139 | CHRNA7 |
| 23152 | CIC |
| 1149 | CIDEA |
| 10370 | CITED2 |
| 1152 | CKB |
| 1158 | CKM |
| 1164 | CKS2 |
| 1182 | CLCN3 |
| 1184 | CLCN5 |
| 9076 | CLDN1 |
| 1365 | CLDN3 |
| 1364 | CLDN4 |
| 1366 | CLDN7 |
| 9080 | CLDN9 |
| 6320 | CLEC11A |
| 25932 | CLIC4 |
| 1201 | CLN3 |
| 1207 | CLNS1A |
| 1209 | CLPTM1 |
| 1213 | CLTC |
| 1191 | CLU |
| 55783 | CMTR2 |
| 1265 | CNN2 |
| 1266 | CNN3 |
| 4849 | CNOT3 |
| 10330 | CNPY2 |
| 1268 | CNR1 |
| 1269 | CNR2 |
| 26047 | CNTNAP2 |
| 116840 | CNTROB |
| 1301 | COL11A1 |
| 1303 | COL12A1 |
| 80781 | COL18A1 |
| 1280 | COL2A1 |
| 1284 | COL4A2 |
| 10087 | COL4A3BP |
| 1287 | COL4A5 |
| 1288 | COL4A6 |
| 1289 | COL5A1 |
| 1291 | COL6A1 |
| 1294 | COL7A1 |
| 1312 | COMT |
| 10987 | COPS5 |
| 50813 | COPS7A |
| 11151 | CORO1A |
| 23603 | CORO1C |
| 23406 | COTL1 |
| 10063 | COX17 |
| 1356 | CP |
| 27151 | CPAMD8 |
| 64506 | CPEB1 |
| 8895 | CPNE3 |
| 10404 | CPQ |
| 1373 | CPS1 |
| 29894 | CPSF1 |
| 1374 | CPT1A |
| 1375 | CPT1B |
| 126129 | CPT1C |
| 1381 | CRABP1 |
| 8738 | CRADD |
| 1385 | CREB1 |
| 1387 | CREBBP |
| 8804 | CREG1 |
| 1401 | CRP |
| 23373 | CRTC1 |
| 1407 | CRY1 |
| 1408 | CRY2 |
| 1410 | CRYAB |
| 51084 | CRYL1 |
| 1435 | CSF1 |
| 1436 | CSF1R |
| 1437 | CSF2 |
| 1440 | CSF3 |
| 1447 | CSN2 |
| 1452 | CSNK1A1 |
| 1457 | CSNK2A1 |
| 1459 | CSNK2A2 |
| 1460 | CSNK2B |
| 1469 | CST1 |
| 1471 | CST3 |
| 1474 | CST6 |
| 8530 | CST7 |
| 1475 | CSTA |
| 1476 | CSTB |
| 1487 | CTBP1 |
| 1488 | CTBP2 |
| 10664 | CTCF |
| 1490 | CTGF |
| 1493 | CTLA4 |
| 1495 | CTNNA1 |
| 1496 | CTNNA2 |
| 1499 | CTNNB1 |
| 1501 | CTNND2 |
| 1508 | CTSB |
| 1075 | CTSC |
| 1509 | CTSD |
| 1512 | CTSH |
| 1513 | CTSK |
| 1514 | CTSL |
| 1520 | CTSS |
| 1522 | CTSZ |
| 2017 | CTTN |
| 8029 | CUBN |
| 8452 | CUL3 |
| 8065 | CUL5 |
| 9820 | CUL7 |
| 1523 | CUX1 |
| 6376 | CX3CL1 |
| 3627 | CXCL10 |
| 6387 | CXCL12 |
| 10563 | CXCL13 |
| 2920 | CXCL2 |
| 2921 | CXCL3 |
| 3576 | CXCL8 |
| 4283 | CXCL9 |
| 7852 | CXCR4 |
| 1540 | CYLD |
| 1583 | CYP11A1 |
| 1585 | CYP11B2 |
| 1586 | CYP17A1 |
| 1588 | CYP19A1 |
| 1543 | CYP1A1 |
| 1544 | CYP1A2 |
| 1545 | CYP1B1 |
| 1591 | CYP24A1 |
| 1592 | CYP26A1 |
| 1553 | CYP2A13 |
| 1548 | CYP2A6 |
| 1555 | CYP2B6 |
| 1562 | CYP2C18 |
| 1557 | CYP2C19 |
| 1558 | CYP2C8 |
| 1559 | CYP2C9 |
| 1565 | CYP2D6 |
| 1571 | CYP2E1 |
| 1576 | CYP3A4 |
| 64816 | CYP3A43 |
| 1577 | CYP3A5 |
| 1580 | CYP4B1 |
| 9420 | CYP7B1 |
| 3491 | CYR61 |
| 57105 | CYSLTR2 |
| 153090 | DAB2IP |
| 168002 | DACT2 |
| 1605 | DAG1 |
| 7818 | DAP3 |
| 1612 | DAPK1 |
| 1616 | DAXX |
| 1618 | DAZL |
| 1621 | DBH |
| 1622 | DBI |
| 55827 | DCAF6 |
| 10238 | DCAF7 |
| 117159 | DCD |
| 9201 | DCLK1 |
| 1634 | DCN |
| 51181 | DCXR |
| 1642 | DDB1 |
| 1643 | DDB2 |
| 1644 | DDC |
| 1649 | DDIT3 |
| 54541 | DDIT4 |
| 780 | DDR1 |
| 4921 | DDR2 |
| 1654 | DDX3X |
| 317781 | DDX51 |
| 79039 | DDX54 |
| 50514 | dec-01 |
| 1668 | DEFA3 |
| 1670 | DEFA5 |
| 1672 | DEFB1 |
| 8560 | DEGS1 |
| 7913 | DEK |
| 9681 | DEPDC5 |
| 1674 | DES |
| 51029 | DESI2 |
| 27294 | DHDH |
| 1719 | DHFR |
| 1723 | DHODH |
| 1665 | DHX15 |
| 56616 | DIABLO |
| 23405 | DICER1 |
| 1734 | DIO2 |
| 1735 | DIO3  Bottom of Form |
| 22982 | DIP2C  Top of Form |
| 129563 | DIS3L2 |
| 22943 | DKK1 |
| 10395 | DLC1 |
| 9940 | DLEC1 |
| 1740 | DLG2 |
| 1742 | DLG4 |
| 28514 | DLL1 |
| 54567 | DLL4 |
| 1748 | DLX4 |
| 1755 | DMBT1 |
| 1756 | DMD |
| 1761 | DMRT1 |
| 64215 | DNAJC1 |
| 54431 | DNAJC10 |
| 29103 | DNAJC15 |
| 5611 | DNAJC3 |
| 1775 | DNASE1L2 |
| 1776 | DNASE1L3 |
| 144132 | DNHD1 |
| 1786 | DNMT1 |
| 1788 | DNMT3A |
| 1789 | DNMT3B |
| 1794 | DOCK2 |
| 84444 | DOT1L |
| 1798 | DPAGT1 |
| 57628 | DPP10 |
| 359787 | DPPA3 |
| 1806 | DPYD |
| 10589 | DRAP1 |
| 1813 | DRD2 |
| 1823 | DSC1 |
| 1825 | DSC3 |
| 51514 | DTL |
| 1840 | DTX1 |
| 113878 | DTX2 |
| 196403 | DTX3 |
| 23220 | DTX4 |
| 1845 | DUSP3 |
| 1848 | DUSP6 |
| 100288687 | DUX4 |
| 79659 | DYNC2H1 |
| 9149 | DYRK1B |
| 1869 | E2F1 |
| 55840 | EAF2 |
| 1879 | EBF1 |
| 10148 | EBI3 |
| 55862 | ECHDC1 |
| 1892 | ECHS1 |
| 1896 | EDA |
| 1906 | EDN1 |
| 1909 | EDNRA |
| 1910 | EDNRB |
| 8726 | EED |
| 1915 | EEF1A1 |
| 1917 | EEF1A2 |
| 1933 | EEF1B2 |
| 1938 | EEF2 |
| 2202 | EFEMP1 |
| 1942 | EFNA1 |
| 1948 | EFNB2 |
| 1950 | EGF |
| 1956 | EGFR |
| 1958 | EGR1 |
| 1959 | EGR2 |
| 23301 | EHBP1 |
| 30845 | EHD3 |
| 26298 | EHF |
| 79813 | EHMT1 |
| 10919 | EHMT2 |
| 9538 | EI24 |
| 23741 | EID1 |
| 84285 | EIF1AD |
| 1964 | EIF1AX |
| 5610 | EIF2AK2 |
| 8890 | EIF2B4 |
| 8893 | EIF2B5 |
| 1965 | EIF2S1 |
| 8894 | EIF2S2 |
| 8661 | EIF3A |
| 8667 | EIF3H |
| 8668 | EIF3I |
| 1974 | EIF4A2 |
| 1977 | EIF4E |
| 1978 | EIF4EBP1 |
| 1984 | EIF5A |
| 3692 | EIF6 |
| 60528 | ELAC2 |
| 1993 | ELAVL2 |
| 1999 | ELF3 |
| 2004 | ELK3 |
| 9844 | ELMO1 |
| 6921 | ELOC |
| 27436 | EML4 |
| 2014 | EMP3 |
| 56946 | EMSY |
| 2018 | EMX2 |
| 2019 | EN1 |
| 55740 | ENAH |
| 2023 | ENO1 |
| 2026 | ENO2 |
| 10495 | ENOX2 |
| 5168 | ENPP2 |
| 59084 | ENPP5 |
| 955 | ENTPD6 |
| 8320 | EOMES |
| 2033 | EP300 |
| 2034 | EPAS1 |
| 2035 | EPB41 |
| 23136 | EPB41L3 |
| 4072 | EPCAM |
| 2041 | EPHA1 |
| 1969 | EPHA2 |
| 2045 | EPHA7 |
| 2048 | EPHB2 |
| 2050 | EPHB4 |
| 2052 | EPHX1 |
| 79852 | EPHX3 |
| 2056 | EPO |
| 2064 | ERBB2 |
| 2065 | ERBB3 |
| 2066 | ERBB4 |
| 55914 | ERBIN |
| 2067 | ERCC1 |
| 2068 | ERCC2 |
| 2071 | ERCC3 |
| 2072 | ERCC4 |
| 2073 | ERCC5 |
| 2074 | ERCC6 |
| 2069 | EREG |
| 2078 | ERG |
| 10961 | ERP29 |
| 23071 | ERP44 |
| 54206 | ERRFI1 |
| 9700 | ESPL1 |
| 2099 | ESR1 |
| 2100 | ESR2 |
| 2101 | ESRRA |
| 54465 | ETAA1 |
| 2108 | ETFA |
| 2114 | ETS2 |
| 2115 | ETV1 |
| 2118 | ETV4 |
| 2120 | ETV6 |
| 51466 | EVL |
| 2130 | EWSR1 |
| 9156 | EXO1 |
| 23404 | EXOSC2 |
| 56915 | EXOSC5 |
| 2131 | EXT1 |
| 2132 | EXT2 |
| 2070 | EYA4 |
| 2146 | EZH2 |
| 7430 | EZR |
| 2161 | F12 |
| 2147 | F2 |
| 2149 | F2R |
| 2150 | F2RL1 |
| 2152 | F3 |
| 2153 | F5 |
| 2158 | F9 |
| 158584 | FAAH2 |
| 2167 | FABP4 |
| 2171 | FABP5 |
| 2173 | FABP7 |
| 8772 | FADD |
| 3992 | FADS1 |
| 9415 | FADS2 |
| 23197 | FAF2 |
| 23201 | FAM168A |
| 2175 | FANCA |
| 9855 | FARP2 |
| 355 | FAS |
| 356 | FASLG |
| 2194 | FASN |
| 2195 | FAT1 |
| 2196 | FAT2 |
| 2091 | FBL |
| 2192 | FBLN1 |
| 10516 | FBLN5 |
| 2203 | FBP1 |
| 93611 | FBXO44 |
| 55294 | FBXW7 |
| 26259 | FBXW8 |
| 2220 | FCN2 |
| 2222 | FDFT1 |
| 2237 | FEN1 |
| 10979 | FERMT2 |
| 2243 | FGA |
| 221472 | FGD2 |
| 152273 | FGD5 |
| 55785 | FGD6 |
| 2246 | FGF1 |
| 2255 | FGF10 |
| 2258 | FGF13 |
| 8823 | FGF16 |
| 8817 | FGF18 |
| 9965 | FGF19 |
| 2247 | FGF2 |
| 2248 | FGF3 |
| 2249 | FGF4 |
| 2254 | FGF9 |
| 2260 | FGFR1 |
| 2263 | FGFR2 |
| 2261 | FGFR3 |
| 2264 | FGFR4 |
| 2266 | FGG |
| 2271 | FH |
| 2272 | FHIT |
| 2274 | FHL2 |
| 27145 | FILIP1 |
| 2280 | FKBP1A |
| 2286 | FKBP2 |
| 201163 | FLCN |
| 2313 | FLI1 |
| 2316 | FLNA |
| 2318 | FLNC |
| 2321 | FLT1 |
| 2322 | FLT3 |
| 2324 | FLT4 |
| 56776 | FMN2 |
| 2328 | FMO3 |
| 2335 | FN1 |
| 2346 | FOLH1 |
| 2348 | FOLR1 |
| 2353 | FOS |
| 2354 | FOSB |
| 2355 | FOSL2 |
| 3169 | FOXA1 |
| 3171 | FOXA3 |
| 2296 | FOXC1 |
| 2294 | FOXF1 |
| 668 | FOXL2 |
| 2305 | FOXM1 |
| 2308 | FOXO1 |
| 2309 | FOXO3 |
| 27086 | FOXP1 |
| 93986 | FOXP2 |
| 50943 | FOXP3 |
| 116113 | FOXP4 |
| 94234 | FOXQ1 |
| 139628 | FOXR2 |
| 2356 | FPGS |
| 8790 | FPGT |
| 79187 | FSD1 |
| 2492 | FSHR |
| 10468 | FST |
| 10841 | FTCD |
| 2495 | FTH1 |
| 79068 | FTO |
| 5045 | FURIN |
| 2521 | FUS |
| 2529 | FUT7 |
| 2534 | FYN |
| 2535 | FZD2 |
| 50486 | G0S2 |
| 2549 | GAB1 |
| 9846 | GAB2 |
| 1647 | GADD45A |
| 339896 | GADL1 |
| 79695 | GALNT12 |
| 2591 | GALNT3 |
| 2597 | GAPDH |
| 2620 | GAS2 |
| 10634 | GAS2L1 |
| 2621 | GAS6 |
| 2624 | GATA2 |
| 2625 | GATA3 |
| 2626 | GATA4 |
| 140628 | GATA5 |
| 2627 | GATA6 |
| 2628 | GATM |
| 2638 | GC |
| 2641 | GCG |
| 2729 | GCLC |
| 2650 | GCNT1 |
| 9615 | GDA |
| 2662 | GDF10 |
| 9518 | GDF15 |
| 2658 | GDF2 |
| 9573 | GDF3 |
| 2661 | GDF9 |
| 2668 | GDNF |
| 2672 | GFI1 |
| 8836 | GGH |
| 2678 | GGT1 |
| 2688 | GH1 |
| 2690 | GHR |
| 51738 | GHRL |
| 2693 | GHSR |
| 51659 | GINS2 |
| 2696 | GIPR |
| 2697 | GJA1 |
| 2701 | GJA4 |
| 2705 | GJB1 |
| 2706 | GJB2 |
| 2709 | GJB5 |
| 56287 | GKN1 |
| 2717 | GLA |
| 2735 | GLI1 |
| 2736 | GLI2 |
| 2737 | GLI3 |
| 11010 | GLIPR1 |
| 11146 | GLMN |
| 2739 | GLO1 |
| 2740 | GLP1R |
| 29997 | NOP53 |
| 2752 | GLUL |
| 51292 | GMPR2 |
| 8833 | GMPS |
| 2771 | GNAI2 |
| 2776 | GNAQ |
| 2778 | GNAS |
| 2787 | GNG5 |
| 27232 | GNMT |
| 2796 | GNRH1 |
| 2812 | GP1BB |
| 57678 | GPAM |
| 2719 | GPC3 |
| 56261 | GPCPD1 |
| 2852 | GPER1 |
| 170589 | GPHA2 |
| 2821 | GPI |
| 10457 | GPNMB |
| 1880 | GPR183 |
| 2861 | GPR37 |
| 9290 | GPR55 |
| 29899 | GPSM2 |
| 2876 | GPX1 |
| 2877 | GPX2 |
| 2878 | GPX3 |
| 2879 | GPX4 |
| 257202 | GPX6 |
| 2886 | GRB7 |
| 26585 | GREM1 |
| 9380 | GRHPR |
| 2903 | GRIN2A |
| 2911 | GRM1 |
| 2913 | GRM3 |
| 2914 | GRM4 |
| 2918 | GRM8 |
| 2925 | GRPR |
| 2932 | GSK3B |
| 51527 | GSKIP |
| 2934 | GSN |
| 2937 | GSS |
| 2938 | GSTA1 |
| 2941 | GSTA4 |
| 373156 | GSTK1 |
| 2944 | GSTM1 |
| 2946 | GSTM2 |
| 2947 | GSTM3 |
| 2949 | GSTM5 |
| 9446 | GSTO1 |
| 119391 | GSTO2 |
| 2950 | GSTP1 |
| 2952 | N/A |
| 2954 | GSTZ1 |
| 2969 | GTF2I |
| 2977 | GUCY1A2 |
| 2982 | GUCY1A1 |
| 2984 | GUCY2C |
| 3002 | GZMB |
| 3005 | H1F0 |
| 3014 | H2AFX |
| 3020 | H3F3A |
| 3021 | H3F3B |
| 9200 | HACD1 |
| 57531 | HACE1 |
| 3033 | HADH |
| 3032 | HADHB |
| 54363 | HAO1 |
| 3035 | HARS |
| 3039 | HBA1 |
| 1839 | HBEGF |
| 338442 | HCAR2 |
| 3054 | HCFC1 |
| 3055 | HCK |
| 3065 | HDAC1 |
| 3066 | HDAC2 |
| 9759 | HDAC4 |
| 10013 | HDAC6 |
| 3069 | HDLBP |
| 3070 | HELLS |
| 9843 | HEPH |
| 8916 | HERC3 |
| 9709 | HERPUD1 |
| 3280 | HES1 |
| 388585 | HES5 |
| 23462 | HEY1 |
| 23493 | HEY2 |
| 26508 | HEYL |
| 3082 | HGF |
| 3083 | HGFAC |
| 3087 | HHEX |
| 3090 | HIC1 |
| 3091 | HIF1A |
| 192286 | HIGD2A |
| 3092 | HIP1 |
| 3006 | HIST1H1C |
| 3008 | HIST1H1E |
| 8332 | HIST1H2AL |
| 8347 | HIST1H2BC |
| 8339 | HIST1H2BG |
| 85236 | HIST1H2BK |
| 8342 | HIST1H2BM |
| 8358 | HIST1H3B |
| 8365 | HIST1H4H |
| 3105 | HLA-A |
| 3106 | HLA-B |
| 3107 | HLA-C |
| 3115 | HLA-DPB1 |
| 3117 | HLA-DQA2/ HLA-DQA1 |
| 3119 | HLA-DQB1 |
| 3123 | HLA-DRB1 |
| 3131 | HLF |
| 6596 | HLTF |
| 3159 | HMGA1 |
| 8091 | HMGA2 |
| 3146 | HMGB1 |
| 3148 | HMGB2 |
| 3156 | HMGCR |
| 3158 | HMGCS2 |
| 3150 | HMGN1 |
| 3161 | HMMR |
| 3162 | HMOX1 |
| 6927 | HNF1A |
| 6928 | HNF1B |
| 3178 | HNRNPA1 |
| 3181 | HNRNPA2B1 |
| 3182 | HNRNPAB |
| 3187 | HNRNPH1 |
| 3190 | HNRNPK |
| 3191 | HNRNPL |
| 10236 | HNRNPR |
| 84376 | HOOK3 |
| 3206 | HOXA10 |
| 3207 | HOXA11 |
| 3199 | HOXA2 |
| 3202 | HOXA5 |
| 3205 | HOXA9 |
| 10481 | HOXB13 |
| 3231 | HOXD1 |
| 3236 | HOXD10 |
| 3237 | HOXD11 |
| 3238 | HOXD12 |
| 3232 | HOXD3 |
| 3233 | HOXD4 |
| 3235 | HOXD9 |
| 3240 | HP |
| 3248 | HPGD |
| 27306 | HPGDS |
| 3249 | HPN |
| 10855 | HPSE |
| 3265 | HRAS |
| 3273 | HRG |
| 59340 | HRH4 |
| 222537 | HS3ST5 |
| 3292 | HSD17B1 |
| 3028 | HSD17B10 |
| 3294 | HSD17B2 |
| 3293 | HSD17B3 |
| 3283 | HSD3B1 |
| 3284 | HSD3B2 |
| 84263 | HSDL2 |
| 3297 | HSF1 |
| 3320 | HSP90AA1 |
| 3326 | HSP90AB1 |
| 7184 | HSP90B1 |
| 3303 | HSPA1A |
| 3304 | HSPA1B |
| 3306 | HSPA2 |
| 3309 | HSPA5 |
| 3312 | HSPA8 |
| 3313 | HSPA9 |
| 3315 | HSPB1 |
| 3329 | HSPD1 |
| 10553 | HTATIP2 |
| 3350 | HTR1A |
| 55699 | IARS2 |
| 3383 | ICAM1 |
| 7087 | ICAM5 |
| 29851 | ICOS |
| 3397 | ID1 |
| 3398 | ID2 |
| 3399 | ID3 |
| 3400 | ID4 |
| 3417 | IDH1 |
| 3418 | IDH2 |
| 3420 | IDH3B |
| 3620 | IDO1 |
| 10437 | IFI30 |
| 3439 | IFNA1 |
| 3440 | IFNA2 |
| 3442 | IFNA5 |
| 3456 | IFNB1 |
| 3458 | IFNG |
| 8100 | IFT88 |
| 3476 | IGBP1 |
| 3479 | IGF1 |
| 3480 | IGF1R |
| 3481 | IGF2 |
| 10643 | IGF2BP3 |
| 3482 | IGF2R |
| 3484 | IGFBP1 |
| 3485 | IGFBP2 |
| 3486 | IGFBP3 |
| 3488 | IGFBP5 |
| 3489 | IGFBP6 |
| 3490 | IGFBP7 |
| 3500 | N/A |
| 28444 | N/A |
| 150084 | IGSF5 |
| 3549 | IHH |
| 8517 | IKBKG |
| 10320 | IKZF1 |
| 22807 | IKZF2 |
| 22806 | IKZF3 |
| 3586 | IL10 |
| 3592 | IL12A |
| 3593 | IL12B |
| 3596 | IL13 |
| 3603 | IL16 |
| 3605 | IL17A |
| 84818 | IL17RC |
| 54756 | IL17RD |
| 3606 | IL18 |
| 29949 | IL19 |
| 3552 | IL1A |
| 3553 | IL1B |
| 3557 | IL1RN |
| 3558 | IL2 |
| 11009 | IL24 |
| 246778 | IL27 |
| 3559 | IL2RA |
| 3561 | IL2RG |
| 3562 | IL3 |
| 146433 | IL34 |
| 3565 | IL4 |
| 3566 | IL4R |
| 3569 | IL6 |
| 3570 | IL6R |
| 3572 | IL6ST |
| 3575 | IL7R |
| 3611 | ILK |
| 3619 | INCENP |
| 3621 | ING1 |
| 3624 | INHBA |
| 3632 | INPP5A |
| 3630 | INS |
| 3638 | INSIG1 |
| 3642 | INSM1 |
| 3645 | INSRR |
| 92105 | INTS4 |
| 8826 | IQGAP1 |
| 10788 | IQGAP2 |
| 51135 | IRAK4 |
| 3658 | IREB2 |
| 3659 | IRF1 |
| 3660 | IRF2 |
| 359948 | IRF2BP2 |
| 3662 | IRF4 |
| 3664 | IRF6 |
| 3667 | IRS1 |
| 8660 | IRS2 |
| 50805 | IRX4 |
| 3670 | ISL1 |
| 51477 | ISYNA1 |
| 3676 | ITGA4 |
| 3678 | ITGA5 |
| 8516 | ITGA8 |
| 3683 | ITGAL |
| 3684 | ITGAM |
| 3688 | ITGB1 |
| 3689 | ITGB2 |
| 3690 | ITGB3 |
| 3691 | ITGB4 |
| 3697 | ITIH1 |
| 3699 | ITIH3 |
| 3700 | ITIH4 |
| 55600 | ITLN1 |
| 3705 | ITPK1 |
| 3708 | ITPR1 |
| 50618 | ITSN2 |
| 3713 | IVL |
| 10625 | IVNS1ABP |
| 182 | JAG1 |
| 3714 | JAG2 |
| 3717 | JAK2 |
| 3718 | JAK3 |
| 221895 | JAZF1 |
| 122953 | JDP2 |
| 221037 | JMJD1C |
| 3725 | JUN |
| 3726 | JUNB |
| 3728 | JUP |
| 284058 | KANSL1 |
| 7994 | KAT6A |
| 3756 | KCNH1 |
| 3757 | KCNH2 |
| 3762 | KCNJ5  Bottom of Form |
| 3784 | KCNQ1  Top of Form |
| 10945 | KDELR1 |
| 23028 | KDM1A |
| 23030 | KDM4B |
| 23081 | KDM4C |
| 10765 | KDM5B |
| 8242 | KDM5C |
| 7403 | KDM6A |
| 23135 | KDM6B |
| 3791 | KDR |
| 2531 | KDSR |
| 9817 | KEAP1 |
| 202559 | KHDRBS2 |
| 8570 | KHSRP |
| 3832 | KIF11 |
| 23095 | KIF1B |
| 55605 | KIF21A |
| 3799 | KIF5B |
| 3814 | KISS1 |
| 3815 | KIT |
| 4254 | KITLG |
| 28999 | KLF15 |
| 688 | KLF5 |
| 1316 | KLF6 |
| 200942 | KLHDC8B |
| 5655 | KLK10 |
| 3817 | KLK2 |
| 354 | KLK3 |
| 25818 | KLK5 |
| 22914 | KLRK1 |
| 4297 | KMT2A |
| 9757 | KMT2B |
| 58508 | KMT2C |
| 8085 | KMT2D |
| 55904 | KMT2E |
| 3827 | KNG1 |
| 90417 | KNSTRN |
| 3845 | KRAS |
| 889 | KRIT1 |
| 3848 | KRT1 |
| 3858 | KRT10 |
| 3860 | KRT13 |
| 3861 | KRT14 |
| 3866 | KRT15 |
| 3868 | KRT16 |
| 3872 | KRT17 |
| 3875 | KRT18 |
| 3880 | KRT19 |
| 54474 | KRT20 |
| 3850 | KRT3 |
| 3882 | KRT32 |
| 3852 | KRT5 |
| 3854 | KRT6B |
| 3855 | KRT7 |
| 112802 | KRT71 |
| 3856 | KRT8 |
| 883 | KYAT1 |
| 83746 | L3MBTL2 |
| 84456 | L3MBTL3 |
| 3913 | LAMB2 |
| 3915 | LAMC1 |
| 10542 | LAMTOR5 |
| 9215 | LARGE1 |
| 113251 | LARP4 |
| 9113 | LATS1 |
| 26524 | LATS2 |
| 10660 | LBX1 |
| 3934 | LCN2 |
| 60526 | LDAH |
| 92483 | LDHAL6B |
| 3945 | LDHB |
| 51176 | LEF1 |
| 3952 | LEP |
| 3953 | LEPR |
| 3956 | LGALS1 |
| 3958 | LGALS3 |
| 8549 | LGR5 |
| 3972 | LHB |
| 3973 | LHCGR |
| 3976 | LIF |
| 3977 | LIFR |
| 3978 | LIG1 |
| 3981 | LIG4 |
| 23547 | LILRA4 |
| 79727 | LIN28A |
| 389421 | LIN28B |
| 8825 | LIN7A |
| 3996 | LLGL1 |
| 4000 | LMNA |
| 160492 | LMNTD1 |
| 4004 | LMO1 |
| 4005 | LMO2 |
| 22853 | LMTK2 |
| 4012 | LNPEP |
| 4014 | LOR |
| 4015 | LOX |
| 4016 | LOXL1 |
| 4017 | LOXL2 |
| 84171 | LOXL4 |
| 1902 | LPAR1 |
| 54947 | LPCAT2 |
| 4023 | LPL |
| 116844 | LRG1 |
| 53353 | LRP1B |
| 4036 | LRP2 |
| 120892 | LRRK2 |
| 23658 | LSM5 |
| 4046 | LSP1 |
| 51599 | LSR |
| 4049 | LTA |
| 8425 | LTBP4 |
| 4057 | LTF |
| 4058 | LTK |
| 56925 | LXN |
| 4061 | LY6E |
| 23643 | LY96 |
| 4066 | LYL1 |
| 8216 | LZTR1 |
| 11178 | LZTS1 |
| 4074 | M6PR |
| 140733 | MACROD2 |
| 8379 | MAD1L1 |
| 4085 | MAD2L1 |
| 4100 | MAGEA1 |
| 9223 | MAGI1 |
| 9863 | MAGI2 |
| 55110 | MAGOHB |
| 10892 | MALT1 |
| 55534 | MAML3 |
| 4128 | MAOA |
| 4129 | MAOB |
| 5604 | MAP2K1 |
| 5605 | MAP2K2 |
| 5608 | MAP2K6 |
| 5609 | MAP2K7 |
| 4214 | MAP3K1 |
| 4215 | MAP3K3 |
| 4216 | MAP3K4 |
| 4217 | MAP3K5 |
| 6885 | MAP3K7 |
| 1326 | MAP3K8 |
| 4293 | MAP3K9 |
| 5594 | MAPK1 |
| 1432 | MAPK14 |
| 225689 | MAPK15 |
| 5595 | MAPK3 |
| 5599 | MAPK8 |
| 23162 | MAPK8IP3 |
| 5601 | MAPK9 |
| 22924 | MAPRE3 |
| 220972 | MARCH8 |
| 4082 | MARCKS |
| 4139 | MARK1 |
| 2011 | MARK2 |
| 4143 | MAT1A |
| 4149 | MAX |
| 8932 | MBD2 |
| 125997 | MBD3L2 |
| 8720 | MBTPS1 |
| 51360 | MBTPS2 |
| 4157 | MC1R |
| 4158 | MC2R |
| 4163 | MCC |
| 4170 | MCL1 |
| 4171 | MCM2 |
| 4191 | MDH2 |
| 4192 | MDK |
| 4193 | MDM2 |
| 4194 | MDM4 |
| 4199 | ME1 |
| 2122 | MECOM |
| 4204 | MECP2 |
| 5469 | MED1 |
| 9968 | MED12 |
| 80306 | MED28 |
| 4211 | MEIS1 |
| 4221 | MEN1 |
| 4224 | MEP1A |
| 10461 | MERTK |
| 4233 | MET |
| 131965 | METTL6 |
| 4240 | MFGE8 |
| 23269 | MGA |
| 146664 | MGAT5B |
| 4255 | MGMT |
| 100507436 | MICA |
| 54531 | MIER2 |
| 4282 | MIF |
| 9562 | MINPP1 |
| 406894 | N/A |
| 406899 | N/A |
| 406900 | N/A |
| 406902 | N/A |
| 406903 | N/A |
| 406910 | N/A |
| 406913 | N/A |
| 406920 | N/A |
| 406921 | N/A |
| 406933 | N/A |
| 406935 | N/A |
| 406937 | N/A |
| 442892 | N/A |
| 406947 | N/A |
| 406948 | N/A |
| 406949 | N/A |
| 406950 | N/A |
| 574457 | N/A |
| 406961 | N/A |
| 406953 | N/A |
| 574033 | N/A |
| 406966 | N/A |
| 406968 | N/A |
| 406971 | N/A |
| 406979 | N/A |
| 406984 | N/A |
| 406985 | N/A |
| 406988 | N/A |
| 406989 | N/A |
| 574032 | N/A |
| 406991 | N/A |
| 406994 | N/A |
| 406996 | N/A |
| 407006 | N/A |
| 407007 | N/A |
| 407009 | N/A |
| 407013 | N/A |
| 407014 | N/A |
| 407021 | N/A |
| 407029 | N/A |
| 407030 | N/A |
| 407031 | N/A |
| 407034 | N/A |
| 407035 | N/A |
| 407037 | N/A |
| 442901 | N/A |
| 442910 | N/A |
| 407040 | N/A |
| 407042 | N/A |
| 574413 | N/A |
| 494335 | N/A |
| 619552 | N/A |
| 574506 | N/A |
| 407050 | N/A |
| 407053 | N/A |
| 406884 | N/A |
| 406885 | N/A |
| 406891 | N/A |
| 4286 | MITF |
| 4288 | MKI67 |
| 23209 | MLC1 |
| 4291 | MLF1 |
| 4292 | MLH1 |
| 27030 | MLH3 |
| 8028 | MLLT10 |
| 51085 | MLXIPL |
| 4311 | MME |
| 4312 | MMP1 |
| 4319 | MMP10 |
| 4320 | MMP11 |
| 4321 | MMP12 |
| 4322 | MMP13 |
| 4323 | MMP14 |
| 4313 | MMP2 |
| 118856 | MMP21 |
| 10893 | MMP24 |
| 4314 | MMP3 |
| 4316 | MMP7 |
| 4317 | MMP8 |
| 4318 | MMP9 |
| 4330 | MN1 |
| 10933 | MORF4L1 |
| 4353 | MPO |
| 4354 | MPP1 |
| 9902 | MRC2 |
| 28998 | MRPL13 |
| 9801 | MRPL19 |
| 740 | MRPL49 |
| 65005 | MRPL9 |
| 64963 | MRPS11 |
| 28973 | MRPS18B |
| 56945 | MRPS22 |
| 51649 | MRPS23 |
| 28957 | MRPS28 |
| 51081 | MRPS7 |
| 343930 | MSGN1 |
| 4436 | MSH2 |
| 4437 | MSH3 |
| 2956 | MSH6 |
| 10232 | MSLN |
| 4481 | MSR1 |
| 4486 | MST1R |
| 4487 | MSX1 |
| 4495 | MT1G |
| 4504 | MT3 |
| 4507 | MTAP |
| 92140 | MTDH |
| 10797 | MTHFD2 |
| 4524 | MTHFR |
| 9110 | MTMR4 |
| 2475 | MTOR |
| 55149 | MTPAP |
| 4548 | MTR |
| 4552 | MTRR |
| 9788 | MTSS1 |
| 4582 | MUC1 |
| 94025 | MUC16 |
| 4583 | MUC2 |
| 4585 | MUC4 |
| 4588 | MUC6 |
| 4595 | MUTYH |
| 4598 | MVK |
| 4599 | MX1 |
| 4600 | MX2 |
| 4601 | MXI1 |
| 4602 | MYB |
| 4603 | MYBL1 |
| 4609 | MYC |
| 26292 | MYCBP |
| 4610 | MYCL |
| 4613 | MYCN |
| 4615 | MYD88 |
| 4629 | MYH11 |
| 79784 | MYH14 |
| 4626 | MYH8 |
| 4638 | MYLK |
| 84700 | MYO18B |
| 4645 | MYO5B |
| 4647 | MYO7A |
| 4654 | MYOD1 |
| 4665 | NAB2 |
| 55577 | NAGK |
| 4669 | NAGLU |
| 10135 | NAMPT |
| 79923 | NANOG |
| 340719 | NANOS1 |
| 9 | NAT1 |
| 10 | NAT2 |
| 89795 | NAV3 |
| 55739 | NAXD |
| 51594 | NBAS |
| 4683 | NBN |
| 4684 | NCAM1 |
| 1463 | NCAN |
| 653361 | NCF1 |
| 8648 | NCOA1 |
| 10499 | NCOA2 |
| 8202 | NCOA3 |
| 8031 | NCOA4 |
| 9611 | NCOR1 |
| 4692 | NDN |
| 10397 | NDRG1 |
| 57447 | NDRG2 |
| 51079 | NDUFA13 |
| 4695 | NDUFA2 |
| 4719 | NDUFS1 |
| 4722 | NDUFS3 |
| 4723 | NDUFV1 |
| 4745 | NELL1 |
| 9148 | NEURL1 |
| 4763 | NF1 |
| 4771 | NF2 |
| 4780 | NFE2L2 |
| 4781 | NFIB |
| 4782 | NFIC |
| 4790 | NFKB1 |
| 4791 | NFKB2 |
| 4792 | NFKBIA |
| 4800 | NFYA |
| 4803 | NGF |
| 4804 | NGFR |
| 4824 | NKX3-1 |
| 91662 | NLRP12 |
| 199713 | NLRP7 |
| 4830 | NME1 |
| 4831 | NME2 |
| 349565 | NMNAT3 |
| 9397 | NMT2 |
| 4826 | NNAT |
| 10528 | NOP56 |
| 4843 | NOS2 |
| 4846 | NOS3 |
| 4851 | NOTCH1 |
| 4853 | NOTCH2 |
| 4854 | NOTCH3 |
| 4855 | NOTCH4 |
| 27035 | NOX1 |
| 50507 | NOX4 |
| 4869 | NPM1 |
| 4878 | NPPA |
| 4883 | NPR3 |
| 1728 | NQO1 |
| 4835 | NQO2 |
| 8431 | NR0B2 |
| 7376 | NR1H2 |
| 9971 | NR1H4 |
| 8856 | NR1I2 |
| 9970 | NR1I3 |
| 7025 | NR2F1 |
| 2063 | NR2F6 |
| 2908 | NR3C1 |
| 4929 | NR4A2 |
| 8013 | NR4A3 |
| 2516 | NR5A1 |
| 2494 | NR5A2 |
| 441478 | NRARP |
| 4893 | NRAS |
| 4899 | NRF1 |
| 3084 | NRG1 |
| 8204 | NRIP1 |
| 8829 | NRP1 |
| 64324 | NSD1 |
| 7468 | NSD2 |
| 54904 | NSD3 |
| 50814 | NSDHL |
| 26012 | NSMF |
| 30833 | NT5C |
| 22978 | NT5C2 |
| 221294 | NT5DC1 |
| 4907 | NT5E |
| 4913 | NTHL1 |
| 22854 | NTNG1 |
| 4914 | NTRK1 |
| 4915 | NTRK2 |
| 4916 | NTRK3 |
| 9891 | NUAK1 |
| 23386 | NUDCD3 |
| 318 | NUDT2 |
| 283927 | NUDT7 |
| 8021 | NUP214 |
| 4928 | NUP98 |
| 116150 | NUS1 |
| 64359 | NXN |
| 100506658 | OCLN |
| 4953 | ODC1 |
| 4968 | OGG1 |
| 4969 | OGN |
| 10439 | OLFM1 |
| 4978 | OPCML |
| 5008 | OSM |
| 9180 | OSMR |
| 5015 | OTX2 |
| 5017 | OVOL1 |
| 58495 | OVOL2 |
| 5021 | OXTR |
| 5027 | P2RX7 |
| 5034 | P4HB |
| 5036 | PA2G4 |
| 26986 | PABPC1 |
| 23569 | PADI4 |
| 5047 | PAEP |
| 5048 | PAFAH1B1 |
| 5058 | PAK1 |
| 56924 | PAK6 |
| 79728 | PALB2 |
| 23022 | PALLD |
| 8505 | PARG |
| 5071 | PRKN |
| 11315 | PARK7 |
| 142 | PARP1 |
| 143 | PARP4 |
| 10207 | PATJ |
| 5074 | PAWR |
| 5077 | PAX3 |
| 5078 | PAX4 |
| 5079 | PAX5 |
| 5080 | PAX6 |
| 5081 | PAX7 |
| 7849 | PAX8 |
| 55872 | PBK |
| 64081 | PBLD |
| 55193 | PBRM1 |
| 5087 | PBX1 |
| 5091 | PC |
| 5105 | PCK1 |
| 5108 | PCM1 |
| 5111 | PCNA |
| 27344 | PCSK1N |
| 5133 | PDCD1 |
| 27250 | PDCD4 |
| 5138 | PDE2A |
| 5140 | PDE3B |
| 5142 | PDE4B |
| 5144 | PDE4D |
| 8654 | PDE5A |
| 5154 | PDGFA |
| 5155 | PDGFB |
| 56034 | PDGFC |
| 5156 | PDGFRA |
| 5160 | PDHA1 |
| 5162 | PDHB |
| 2923 | PDIA3 |
| 8572 | PDLIM4 |
| 5170 | PDPK1 |
| 10630 | PDPN |
| 3651 | PDX1 |
| 23042 | PDXDC1 |
| 5037 | PEBP1 |
| 5178 | PEG3 |
| 5179 | PENK |
| 5187 | PER1 |
| 8864 | PER2 |
| 8863 | PER3 |
| 5195 | PEX14 |
| 5216 | PFN1 |
| 5223 | PGAM1 |
| 5224 | PGAM2 |
| 5226 | PGD |
| 5230 | PGK1 |
| 5241 | PGR |
| 5245 | PHB |
| 1911 | PHC1 |
| 84295 | PHF6 |
| 26227 | PHGDH |
| 7262 | PHLDA2 |
| 23239 | PHLPP1 |
| 8929 | PHOX2B |
| 10401 | PIAS3 |
| 8301 | PICALM |
| 55367 | PIDD1 |
| 51227 | PIGP |
| 5287 | PIK3C2B |
| 5290 | PIK3CA |
| 5291 | PIK3CB |
| 5293 | PIK3CD |
| 5294 | PIK3CG |
| 5295 | PIK3R1 |
| 5292 | PIM1 |
| 11040 | PIM2 |
| 5300 | PIN1 |
| 65018 | PINK1 |
| 5305 | PIP4K2A |
| 5307 | PITX1 |
| 5308 | PITX2 |
| 5310 | PKD1 |
| 5314 | PKHD1 |
| 5315 | PKM |
| 9088 | PKMYT1 |
| 5317 | PKP1 |
| 11187 | PKP3 |
| 5320 | PLA2G2A |
| 5321 | PLA2G4A |
| 8398 | PLA2G6 |
| 5324 | PLAG1 |
| 5325 | PLAGL1 |
| 5328 | PLAU |
| 5329 | PLAUR |
| 79887 | PLBD1 |
| 23236 | PLCB1 |
| 5332 | PLCB4 |
| 51196 | PLCE1 |
| 5335 | PLCG1 |
| 26499 | PLEK2 |
| 79156 | PLEKHF1 |
| 57449 | PLEKHG5 |
| 123 | PLIN2 |
| 5347 | PLK1 |
| 10769 | PLK2 |
| 5352 | PLOD2 |
| 8611 | PLPP1 |
| 5357 | PLS1 |
| 57088 | PLSCR4 |
| 5366 | PMAIP1 |
| 6490 | PMEL |
| 5371 | PML |
| 5395 | PMS2 |
| 25953 | PNKD |
| 57104 | PNPLA2 |
| 87178 | PNPT1 |
| 5420 | PODXL |
| 5423 | POLB |
| 5424 | POLD1 |
| 10714 | POLD3 |
| 5426 | POLE |
| 5429 | POLH |
| 51426 | POLK |
| 51728 | POLR3K |
| 5443 | POMC |
| 5444 | PON1 |
| 10775 | POP4 |
| 5447 | POR |
| 25913 | POT1 |
| 5457 | POU4F1 |
| 5460 | POU5F1 |
| 11281 | POU6F2 |
| 5465 | PPARA |
| 5467 | PPARD |
| 5468 | PPARG |
| 10891 | PPARGC1A |
| 133522 | PPARGC1B |
| 5471 | PPAT |
| 5478 | PPIA |
| 5480 | PPIC |
| 8493 | PPM1D |
| 22843 | PPM1E |
| 5501 | PPP1CC |
| 10848 | PPP1R13L |
| 94274 | PPP1R14A |
| 26472 | PPP1R14B |
| 5502 | PPP1R1A |
| 5518 | PPP2R1A  Bottom of Form |
| 5527 | PPP2R5C  Top of Form |
| 5530 | PPP3CA |
| 5537 | PPP6C |
| 130814 | PQLC3 |
| 23532 | PRAME |
| 9055 | PRC1 |
| 5546 | PRCC |
| 63978 | PRDM14 |
| 7799 | PRDM2 |
| 5052 | PRDX1 |
| 7001 | PRDX2 |
| 10935 | PRDX3 |
| 10549 | PRDX4 |
| 25824 | PRDX5 |
| 9588 | PRDX6 |
| 5550 | PREP |
| 5551 | PRF1 |
| 5562 | PRKAA1 |
| 5564 | PRKAB1 |
| 5566 | PRKACA |
| 5567 | PRKACB |
| 5573 | PRKAR1A |
| 5578 | PRKCA |
| 5579 | PRKCB |
| 5580 | PRKCD |
| 5581 | PRKCE |
| 5588 | PRKCQ |
| 5589 | PRKCSH |
| 5590 | PRKCZ |
| 5587 | PRKD1 |
| 25865 | PRKD2 |
| 5591 | PRKDC |
| 5617 | PRL |
| 5618 | PRLR |
| 5621 | PRNP |
| 5629 | PROX1 |
| 27339 | PRPF19 |
| 54458 | PRR13 |
| 5396 | PRRX1 |
| 11098 | PRSS23 |
| 5646 | PRSS3 |
| 5652 | PRSS8 |
| 56952 | PRTFDC1 |
| 5657 | PRTN3 |
| 29968 | PSAT1 |
| 8000 | PSCA |
| 11168 | PSIP1 |
| 5685 | PSMA4 |
| 5690 | PSMB2 |
| 29893 | PSMC3IP |
| 5720 | PSME1 |
| 5721 | PSME2 |
| 5724 | PTAFR |
| 5725 | PTBP1 |
| 5727 | PTCH1 |
| 5728 | PTEN |
| 9317 | PTER |
| 5733 | PTGER3 |
| 5734 | PTGER4 |
| 80142 | PTGES2 |
| 5740 | PTGIS |
| 5742 | PTGS1 |
| 5743 | PTGS2 |
| 5745 | PTH1R |
| 5744 | PTHLH |
| 5747 | PTK2 |
| 2185 | PTK2B |
| 5757 | PTMA |
| 5764 | PTN |
| 53635 | PTOV1 |
| 11156 | PTP4A3 |
| 5524 | PTPA |
| 5770 | PTPN1 |
| 5781 | PTPN11 |
| 5784 | PTPN14 |
| 5771 | PTPN2 |
| 5787 | PTPRB |
| 5789 | PTPRD |
| 5792 | PTPRF |
| 5793 | PTPRG |
| 5795 | PTPRJ |
| 5796 | PTPRK |
| 5800 | PTPRO |
| 51651 | PTRH2 |
| 22827 | PUF60 |
| 80324 | PUS1 |
| 7837 | PXDN |
| 5829 | PXN |
| 29108 | PYCARD |
| 5834 | PYGB |
| 5836 | PYGL |
| 5859 | QARS |
| 9444 | QKI |
| 25797 | QPCT |
| 5862 | RAB2A |
| 5864 | RAB3A |
| 53916 | RAB4B |
| 5879 | RAC1 |
| 5880 | RAC2 |
| 29127 | RACGAP1 |
| 10399 | RACK1 |
| 5885 | RAD21 |
| 5886 | RAD23A |
| 5887 | RAD23B |
| 5888 | RAD51 |
| 5890 | RAD51B |
| 5889 | RAD51C |
| 5892 | RAD51D |
| 8438 | RAD54L |
| 5883 | RAD9A |
| 5894 | RAF1 |
| 10743 | RAI1 |
| 10928 | RALBP1 |
| 10266 | RAMP2 |
| 5901 | RAN |
| 5905 | RANGAP1 |
| 5914 | RARA |
| 5915 | RARB |
| 5918 | RARRES1 |
| 5921 | RASA1 |
| 5922 | RASA2 |
| 51655 | RASD1 |
| 5924 | RASGRF2 |
| 10125 | RASGRP1 |
| 11186 | RASSF1 |
| 9770 | RASSF2 |
| 83593 | RASSF5 |
| 30062 | RAX |
| 5925 | RB1 |
| 9821 | RB1CC1 |
| 23543 | RBFOX2 |
| 5934 | RBL2 |
| 5935 | RBM3 |
| 10179 | RBM7 |
| 5947 | RBP1 |
| 5950 | RBP4 |
| 5954 | RCN1 |
| 23186 | RCOR1 |
| 9985 | REC8 |
| 8434 | RECK |
| 5965 | RECQL |
| 5966 | REL |
| 5970 | RELA |
| 5978 | REST |
| 5979 | RET |
| 57455 | REXO1 |
| 5981 | RFC1 |
| 55312 | RFK |
| 55159 | RFWD3 |
| 222546 | RFX6 |
| 9104 | RGN |
| 5997 | RGS2 |
| 6009 | RHEB |
| 387 | RHOA |
| 9699 | RIMS2 |
| 9610 | RIN1 |
| 84864 | RIOX2 |
| 51132 | RLIM |
| 6019 | RLN2 |
| 6038 | RNASE4 |
| 6041 | RNASEL |
| 8635 | RNASET2 |
| 27246 | RNF115 |
| 55819 | RNF130 |
| 11236 | RNF139 |
| 221687 | RNF182 |
| 388591 | RNF207 |
| 80196 | RNF34 |
| 54894 | RNF43 |
| 6049 | RNF6 |
| 6051 | RNPEP |
| 6091 | ROBO1 |
| 6095 | RORA |
| 6098 | ROS1 |
| 6102 | RP2 |
| 6134 | RPL10 |
| 6136 | RPL12 |
| 6137 | RPL13 |
| 9045 | RPL14 |
| 6138 | RPL15 |
| 6141 | RPL18 |
| 6147 | RPL23A |
| 6122 | RPL3 |
| 6160 | RPL31 |
| 6168 | RPL37A |
| 6123 | RPL3L |
| 6125 | RPL5 |
| 6128 | RPL6 |
| 6185 | RPN2 |
| 10557 | RPP38 |
| 6208 | RPS14 |
| 6209 | RPS15 |
| 6223 | RPS19 |
| 6227 | RPS21 |
| 6231 | RPS26 |
| 6188 | RPS3 |
| 6191 | RPS4X |
| 6194 | RPS6 |
| 6195 | RPS6KA1 |
| 6197 | RPS6KA3 |
| 6198 | RPS6KB1 |
| 6199 | RPS6KB2 |
| 6201 | RPS7 |
| 6202 | RPS8 |
| 3921 | RPSA |
| 64121 | RRAGC |
| 6237 | RRAS |
| 6240 | RRM1 |
| 6241 | RRM2 |
| 9136 | RRP9 |
| 284654 | RSPO1 |
| 51750 | RTEL1 |
| 861 | RUNX1 |
| 862 | RUNX1T1 |
| 860 | RUNX2 |
| 864 | RUNX3 |
| 6256 | RXRA |
| 6257 | RXRB |
| 6262 | RYR2 |
| 6277 | S100A6 |
| 6279 | S100A8 |
| 6280 | S100A9 |
| 1901 | S1PR1 |
| 9294 | S1PR2 |
| 29901 | SAC3D1 |
| 27164 | SALL3 |
| 79685 | SAP30L |
| 23314 | SATB2 |
| 22937 | SCAP |
| 949 | SCARB1 |
| 6319 | SCD |
| 6447 | SCG5 |
| 10389 | SCML2 |
| 9805 | SCRN1 |
| 6343 | SCT |
| 6382 | SDC1 |
| 6385 | SDC4 |
| 23753 | SDF2L1 |
| 54949 | SDHAF2 |
| 6390 | SDHB |
| 6391 | SDHC |
| 6392 | SDHD |
| 11231 | SEC63 |
| 6414 | SELENOP |
| 55829 | SELENOS |
| 346288 | sep-14 |
| 10801 | sep-09 |
| 26135 | SERBP1 |
| 10955 | SERINC3 |
| 5265 | SERPINA1 |
| 12 | SERPINA3 |
| 5267 | SERPINA4 |
| 5273 | SERPINB10 |
| 5055 | SERPINB2 |
| 6317 | SERPINB3 |
| 5268 | SERPINB5 |
| 5054 | SERPINE1 |
| 5176 | SERPINF1 |
| 29950 | SERTAD1 |
| 143686 | SESN3 |
| 6418 | SET |
| 29072 | SETD2 |
| 9869 | SETDB1 |
| 7536 | SF1 |
| 23451 | SF3B1 |
| 2810 | SFN |
| 6422 | SFRP1 |
| 6423 | SFRP2 |
| 6424 | SFRP4 |
| 6425 | SFRP5 |
| 6439 | SFTPB |
| 6440 | SFTPC |
| 6441 | SFTPD |
| 6446 | SGK1 |
| 10019 | SH2B3 |
| 6455 | SH3GL1 |
| 30011 | SH3KBP1 |
| 6469 | SHH |
| 6470 | SHMT1 |
| 6472 | SHMT2 |
| 6492 | SIM1 |
| 23411 | SIRT1 |
| 23410 | SIRT3 |
| 6496 | SIX3 |
| 8631 | SKAP1 |
| 6502 | SKP2 |
| 4891 | SLC11A2 |
| 6566 | SLC16A1 |
| 6573 | SLC19A1 |
| 6506 | SLC1A2 |
| 6581 | SLC22A3 |
| 6584 | SLC22A5 |
| 1468 | SLC25A10 |
| 9154 | SLC28A1 |
| 2030 | SLC29A1 |
| 6513 | SLC2A1 |
| 6514 | SLC2A2 |
| 6515 | SLC2A3 |
| 6518 | SLC2A5 |
| 1317 | SLC31A1 |
| 10568 | SLC34A2 |
| 153129 | SLC38A9 |
| 57181 | SLC39A10 |
| 55630 | SLC39A4 |
| 6520 | SLC3A2 |
| 30061 | SLC40A1 |
| 51151 | SLC45A2 |
| 113278 | SLC52A3 |
| 6526 | SLC5A3 |
| 6528 | SLC5A5 |
| 160728 | SLC5A8 |
| 23657 | SLC7A11 |
| 8140 | SLC7A5 |
| 9353 | SLIT2 |
| 4088 | SMAD3 |
| 4089 | SMAD4 |
| 4092 | SMAD7 |
| 6595 | SMARCA2 |
| 6597 | SMARCA4 |
| 6598 | SMARCB1 |
| 6599 | SMARCC1 |
| 6605 | SMARCE1 |
| 8243 | SMC1A |
| 9126 | SMC3 |
| 6608 | SMO |
| 6609 | SMPD1 |
| 55512 | SMPD3 |
| 114826 | SMYD4 |
| 6615 | SNAI1 |
| 6591 | SNAI2 |
| 6623 | SNCG |
| 27044 | SND1 |
| 6628 | SNRPB |
| 6634 | SNRPD3 |
| 27131 | SNX5 |
| 8651 | SOCS1 |
| 9021 | SOCS3 |
| 6647 | SOD1 |
| 6648 | SOD2 |
| 6649 | SOD3 |
| 6651 | SON |
| 6653 | SORL1 |
| 6654 | SOS1 |
| 6664 | SOX11 |
| 6665 | SOX15 |
| 64321 | SOX17 |
| 6657 | SOX2 |
| 11063 | SOX30 |
| 6659 | SOX4 |
| 6662 | SOX9 |
| 6667 | SP1 |
| 11262 | SP140 |
| 389058 | SP5 |
| 6678 | SPARC |
| 25803 | SPDEF |
| 23111 | SPART |
| 8877 | SPHK1 |
| 6688 | SPI1 |
| 6690 | SPINK1 |
| 11005 | SPINK5 |
| 10653 | SPINT2 |
| 6695 | SPOCK1 |
| 10417 | SPON2 |
| 8405 | SPOP |
| 6696 | SPP1 |
| 161742 | SPRED1 |
| 6698 | SPRR1A |
| 6700 | SPRR2A |
| 83932 | SPRTN |
| 10253 | SPRY2 |
| 81848 | SPRY4 |
| 57731 | SPTBN4 |
| 84654 | SPZ1 |
| 6713 | SQLE |
| 6714 | SRC |
| 10847 | SRCAP |
| 6715 | SRD5A1 |
| 6716 | SRD5A2 |
| 6720 | SREBF1 |
| 6721 | SREBF2 |
| 6722 | SRF |
| 6725 | SRMS |
| 6426 | SRSF1 |
| 10772 | SRSF10 |
| 6427 | SRSF2 |
| 6760 | SS18 |
| 23635 | SSBP2 |
| 6750 | SST |
| 6751 | SSTR1 |
| 6752 | SSTR2 |
| 6753 | SSTR3 |
| 6755 | SSTR5 |
| 6756 | SSX1 |
| 6757 | SSX2 |
| 6768 | ST14 |
| 6484 | ST3GAL4 |
| 10735 | STAG2 |
| 6770 | STAR |
| 10809 | STARD10 |
| 10948 | STARD3 |
| 6772 | STAT1 |
| 6774 | STAT3 |
| 6775 | STAT4 |
| 6776 | STAT5A |
| 6777 | STAT5B |
| 6778 | STAT6 |
| 8614 | STC2 |
| 79689 | STEAP4 |
| 6794 | STK11 |
| 8859 | STK19 |
| 3925 | STMN1 |
| 92335 | STRADA |
| 11171 | STRAP |
| 55014 | STX17 |
| 8802 | SUCLG1 |
| 51684 | SUFU |
| 79783 | SUGCT |
| 23213 | SULF1 |
| 6817 | SULT1A1 |
| 6783 | SULT1E1 |
| 6822 | SULT2A1 |
| 6820 | SULT2B1 |
| 7341 | SUMO1 |
| 23512 | SUZ12 |
| 6840 | SVIL |
| 6847 | SYCP1 |
| 6850 | SYK |
| 8189 | SYMPK |
| 23345 | SYNE1 |
| 23224 | SYNE2 |
| 8871 | SYNJ2 |
| 171024 | SYNPO2 |
| 6855 | SYP |
| 6860 | SYT4 |
| 6862 | TBXT |
| 10454 | TAB1 |
| 10460 | TACC3 |
| 8148 | TAF15 |
| 6886 | TAL1 |
| 6888 | TALDO1 |
| 10010 | TANK |
| 83940 | TATDN1 |
| 1155 | TBCB |
| 79718 | TBL1XR1 |
| 6899 | TBX1 |
| 6913 | TBX15 |
| 6926 | TBX3 |
| 6916 | TBXAS1 |
| 6919 | TCEA2 |
| 56849 | TCEAL7 |
| 6939 | TCF15 |
| 6941 | TCF19 |
| 6929 | TCF3 |
| 6925 | TCF4 |
| 83439 | TCF7L1 |
| 6934 | TCF7L2 |
| 9623 | TCL1B |
| 6948 | TCN2 |
| 6950 | TCP1 |
| 8463 | TEAD2 |
| 7010 | TEK |
| 7011 | TEP1 |
| 7014 | TERF2 |
| 7015 | TERT |
| 26136 | TES |
| 54790 | TET2 |
| 7018 | TF |
| 7019 | TFAM |
| 7020 | TFAP2A |
| 7022 | TFAP2C |
| 7029 | TFDP2 |
| 7030 | TFE3 |
| 7031 | TFF1 |
| 7980 | TFPI2 |
| 7037 | TFRC |
| 7039 | TGFA |
| 7040 | TGFB1 |
| 7042 | TGFB2 |
| 7046 | TGFBR1 |
| 7048 | TGFBR2 |
| 7050 | TGIF1 |
| 7052 | TGM2 |
| 7054 | TH |
| 7057 | THBS1 |
| 117145 | THEM4 |
| 9473 | THEMIS2 |
| 7068 | THRB |
| 7069 | THRSP |
| 7076 | TIMP1 |
| 7078 | TIMP3 |
| 7079 | TIMP4 |
| 25976 | TIPARP |
| 114609 | TIRAP |
| 27134 | TJP3 |
| 7083 | TK1 |
| 7086 | TKT |
| 7090 | TLE3 |
| 7091 | TLE4 |
| 9874 | TLK1 |
| 7094 | TLN1 |
| 7097 | TLR2 |
| 7099 | TLR4 |
| 7100 | TLR5 |
| 10333 | TLR6 |
| 3195 | TLX1 |
| 23671 | TMEFF2 |
| 55654 | TMEM127 |
| 121256 | TMEM132D |
| 340061 | TMEM173 |
| 9725 | TMEM63A |
| 7110 | TMF1 |
| 7111 | TMOD1 |
| 7113 | TMPRSS2 |
| 7114 | TMSB4X |
| 3371 | TNC |
| 7124 | TNF |
| 7128 | TNFAIP3 |
| 25816 | TNFAIP8 |
| 8797 | TNFRSF10A |
| 8795 | TNFRSF10B |
| 8792 | TNFRSF11A |
| 23495 | TNFRSF13B |
| 55504 | TNFRSF19 |
| 7133 | TNFRSF1B |
| 27242 | TNFRSF21 |
| 943 | TNFRSF8 |
| 3604 | TNFRSF9 |
| 8743 | TNFSF10 |
| 8600 | TNFSF11 |
| 8741 | TNFSF13 |
| 8744 | TNFSF9 |
| 10318 | TNIP1 |
| 10188 | TNK2 |
| 23112 | TNRC6B |
| 10140 | TOB1 |
| 10040 | TOM1L1 |
| 4796 | TONSL |
| 7150 | TOP1 |
| 7153 | TOP2A |
| 7155 | TOP2B |
| 11073 | TOPBP1 |
| 10210 | TOPORS |
| 27324 | TOX3 |
| 7157 | TP53 |
| 9540 | TP53I3 |
| 8626 | TP63 |
| 7161 | TP73 |
| 7163 | TPD52 |
| 7164 | TPD52L1 |
| 7167 | TPI1 |
| 7168 | TPM1 |
| 7169 | TPM2 |
| 7170 | TPM3 |
| 7171 | TPM4 |
| 1200 | TPP1 |
| 7175 | TPR |
| 7178 | TPT1 |
| 7185 | TRAF1 |
| 84231 | TRAF7 |
| 10131 | TRAP1 |
| 55809 | TRERF1 |
| 7200 | TRH |
| 8805 | TRIM24 |
| 51592 | TRIM33 |
| 7204 | TRIO |
| 54802 | TRIT1 |
| 54822 | TRPM7 |
| 7227 | TRPS1 |
| 7442 | TRPV1 |
| 55503 | TRPV6 |
| 8295 | TRRAP |
| 7248 | TSC1 |
| 7249 | TSC2 |
| 8848 | TSC22D1 |
| 7253 | TSHR |
| 64061 | TSPYL2 |
| 7263 | TST |
| 7270 | TTF1 |
| 7272 | TTK |
| 164395 | TTLL9 |
| 7276 | TTR |
| 84790 | TUBA1C |
| 7280 | TUBB2A |
| 347733 | TUBB2B |
| 10381 | TUBB3 |
| 7284 | TUFM |
| 7991 | TUSC3 |
| 5756 | TWF1 |
| 7291 | TWIST1 |
| 7295 | TXN |
| 84817 | TXNDC17 |
| 81567 | TXNDC5 |
| 10628 | TXNIP |
| 7296 | TXNRD1 |
| 10587 | TXNRD2 |
| 1890 | TYMP |
| 7298 | TYMS |
| 7299 | TYR |
| 7306 | TYRP1 |
| 7317 | UBA1 |
| 7318 | UBA7 |
| 10537 | UBD |
| 11065 | UBE2C |
| 7328 | UBE2H |
| 7329 | UBE2I |
| 29979 | UBQLN1 |
| 51035 | UBXN1 |
| 7345 | UCHL1 |
| 7347 | UCHL3 |
| 7371 | UCK2 |
| 7351 | UCP2 |
| 7352 | UCP3 |
| 7358 | UGDH |
| 54658 | UGT1A1 |
| 7365 | UGT2B10 |
| 7366 | UGT2B15 |
| 7367 | UGT2B17 |
| 7364 | UGT2B7 |
| 29128 | UHRF1 |
| 80328 | ULBP2 |
| 9706 | ULK2 |
| 54986 | ULK4 |
| 7372 | UMPS |
| 7374 | UNG |
| 10975 | UQCR11 |
| 8725 | URI1 |
| 81605 | URM1 |
| 7389 | UROD |
| 11274 | USP18 |
| 9099 | USP2 |
| 7874 | USP7 |
| 9101 | USP8 |
| 57654 | UVSSA |
| 8409 | UXT |
| 6845 | VAMP7 |
| 8673 | VAMP8 |
| 7409 | VAV1 |
| 10451 | VAV3 |
| 7412 | VCAM1 |
| 1462 | VCAN |
| 7421 | VDR |
| 7422 | VEGFA |
| 7423 | VEGFB |
| 7424 | VEGFC |
| 2277 | VEGFD |
| 245806 | VGLL2 |
| 7428 | VHL |
| 7431 | VIM |
| 7432 | VIP |
| 81552 | VOPP1 |
| 23339 | VPS39 |
| 6293 | VPS52 |
| 11326 | VSIG4 |
| 143187 | VTI1A |
| 55717 | WDR11 |
| 9277 | WDR46 |
| 284403 | WDR62 |
| 79084 | WDR77 |
| 7465 | WEE1 |
| 58189 | WFDC1 |
| 80326 | WNT10A |
| 7480 | WNT10B |
| 7472 | WNT2 |
| 7473 | WNT3 |
| 54361 | WNT4 |
| 7474 | WNT5A |
| 7476 | WNT7A |
| 7490 | WT1 |
| 23286 | WWC1 |
| 51741 | WWOX |
| 54739 | XAF1 |
| 7494 | XBP1 |
| 7498 | XDH |
| 331 | XIAP |
| 7507 | XPA |
| 7508 | XPC |
| 7515 | XRCC1 |
| 7516 | XRCC2 |
| 7517 | XRCC3 |
| 10413 | YAP1 |
| 4904 | YBX1 |
| 8089 | YEATS4 |
| 7531 | YWHAE |
| 7534 | YWHAZ |
| 7528 | YY1 |
| 9189 | ZBED1 |
| 7704 | ZBTB16 |
| 26137 | ZBTB20 |
| 51341 | ZBTB7A |
| 339487 | ZBTB8OS |
| 9877 | ZBED6/ ZC3H11A |
| 51201 | ZDHHC2 |
| 6935 | ZEB1 |
| 9839 | ZEB2 |
| 463 | ZFHX3 |
| 7538 | ZFP36 |
| 678 | ZFP36L2 |
| 7546 | ZIC2 |
| 23619 | ZIM2 |
| 80317 | ZKSCAN3 |
| 57178 | ZMIZ1 |
| 9203 | ZMYM3 |
| 9205 | ZMYM5 |
| 90338 | ZNF160 |
| 7730 | ZNF177 |
| 7755 | ZNF205 |
| 7571 | ZNF23 |
| 23036 | ZNF292 |
| 22891 | ZNF365 |
| 167465 | ZNF366 |
| 80264 | ZNF430 |
| 9668 | ZNF432 |
| 220929 | ZNF438 |
| 9745 | ZNF536 |
| 84527 | ZNF559 |
| 148266 | ZNF569 |
| 51042 | ZNF593 |
| 79759 | ZNF668 |
| 91661 | ZNF765 |
| 91752 | ZNF804A |
| 128611 | ZNF831 |
| 30834 | ZNRD1 |
| 84133 | ZNRF3 |
| 8233 | ZRSR2 |
| 342945 | ZSCAN22 |
| 9183 | ZW10 |
| 8745 | ADAM23 |
