# Supplementary figures and images for: The Impact of Immune Interventions: A Systems Biology Strategy for Predicting Adverse and Beneficial Immune Effects
Source: Front Immunol. 2019 Feb 15;10:231. doi: 10.3389/fimmu.2019.00231 (PMC6384242; doi:10.3389/fimmu.2019.00231)

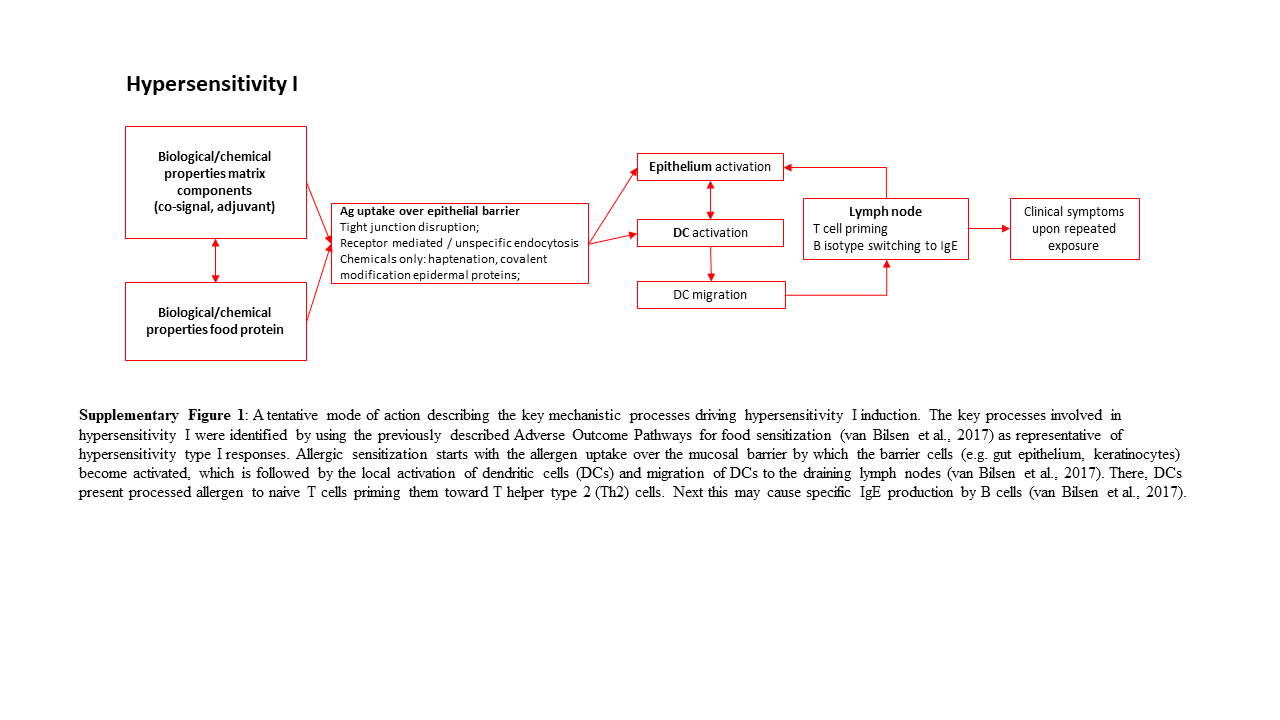

Supplement: Supplementary file 5 [file Image_1.TIF]

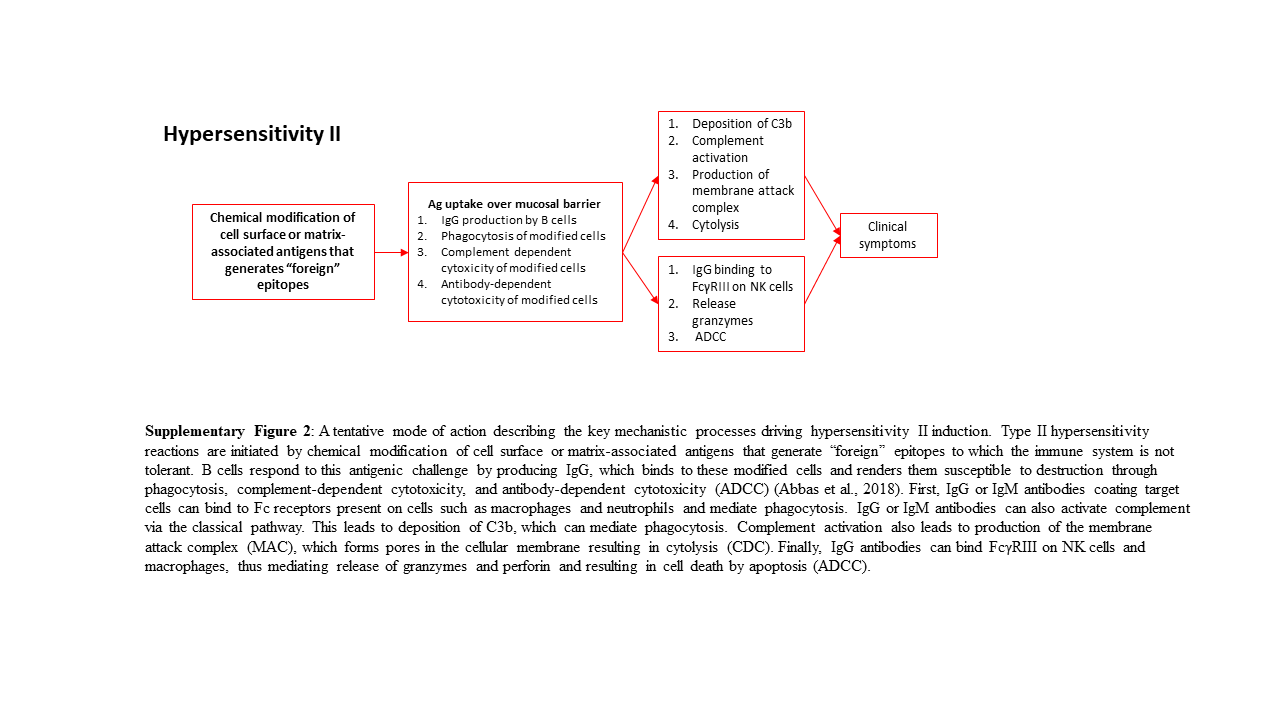

Supplement: Supplementary file 6 [file Image_2.tif]

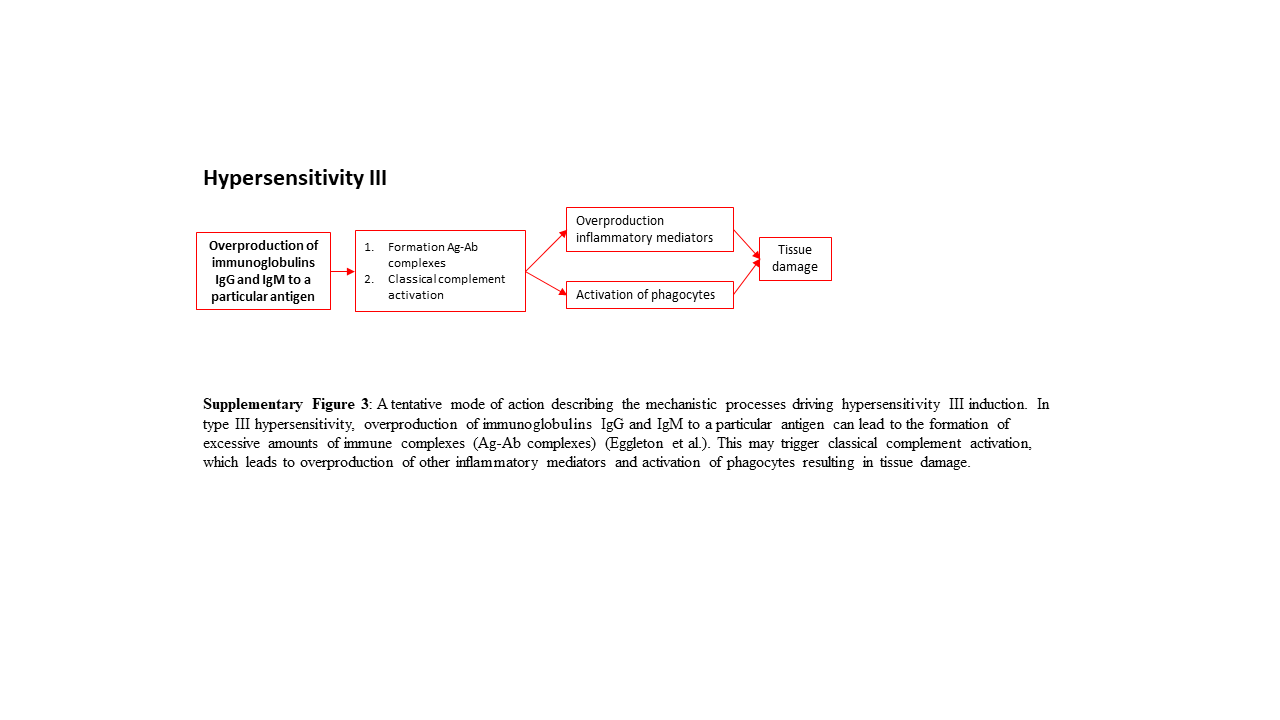

Supplement: Supplementary file 7 [file Image_3.tif]

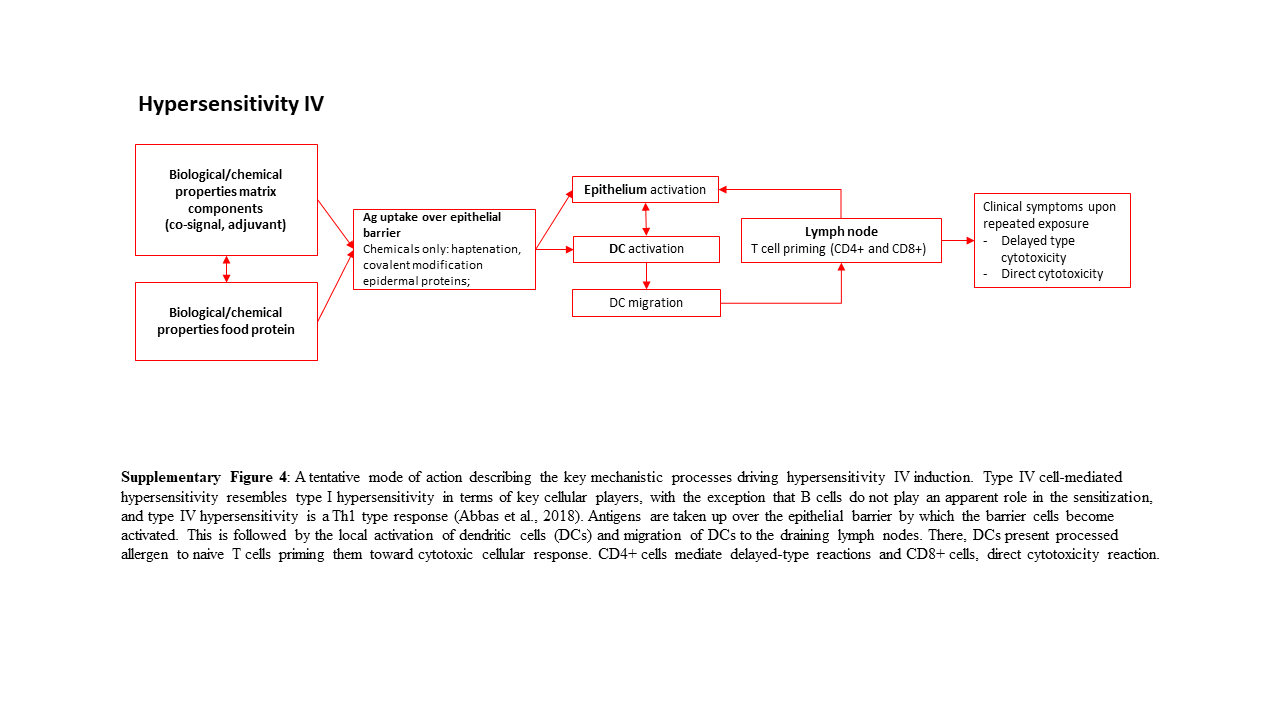

Supplement: Supplementary file 8 [file Image_4.tif]

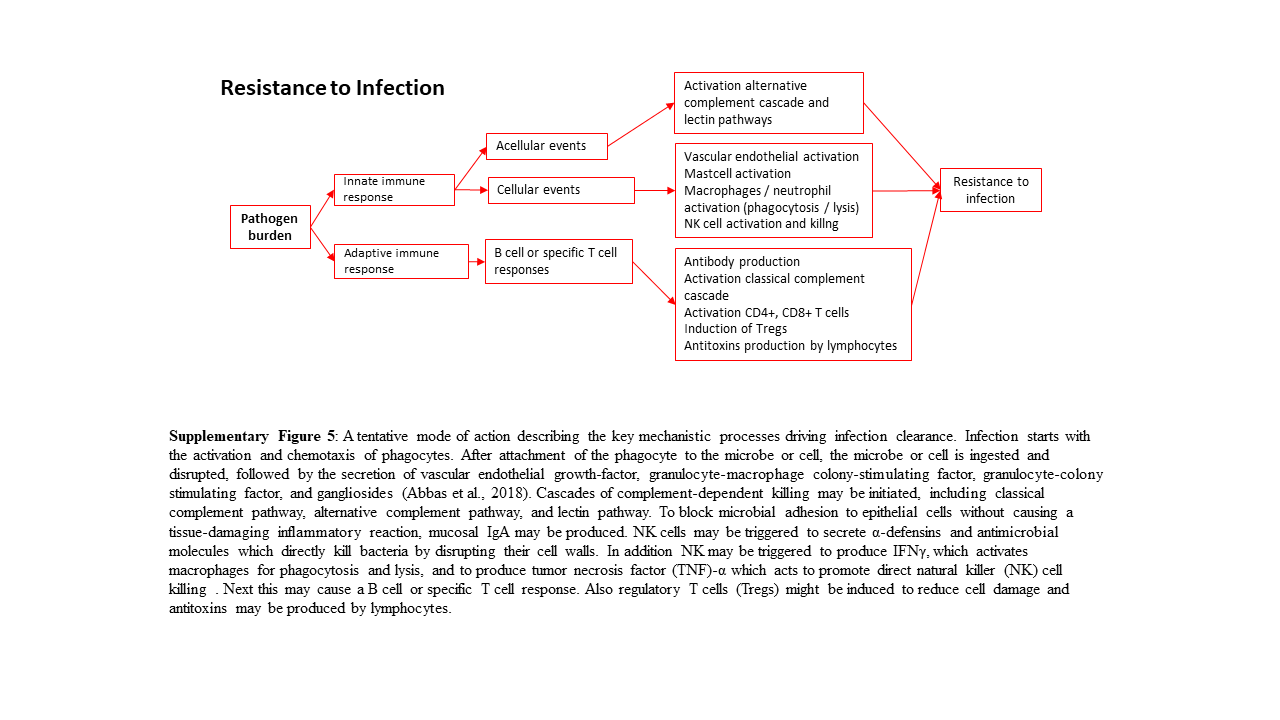

Supplement: Supplementary file 9 [file Image_5.tif]

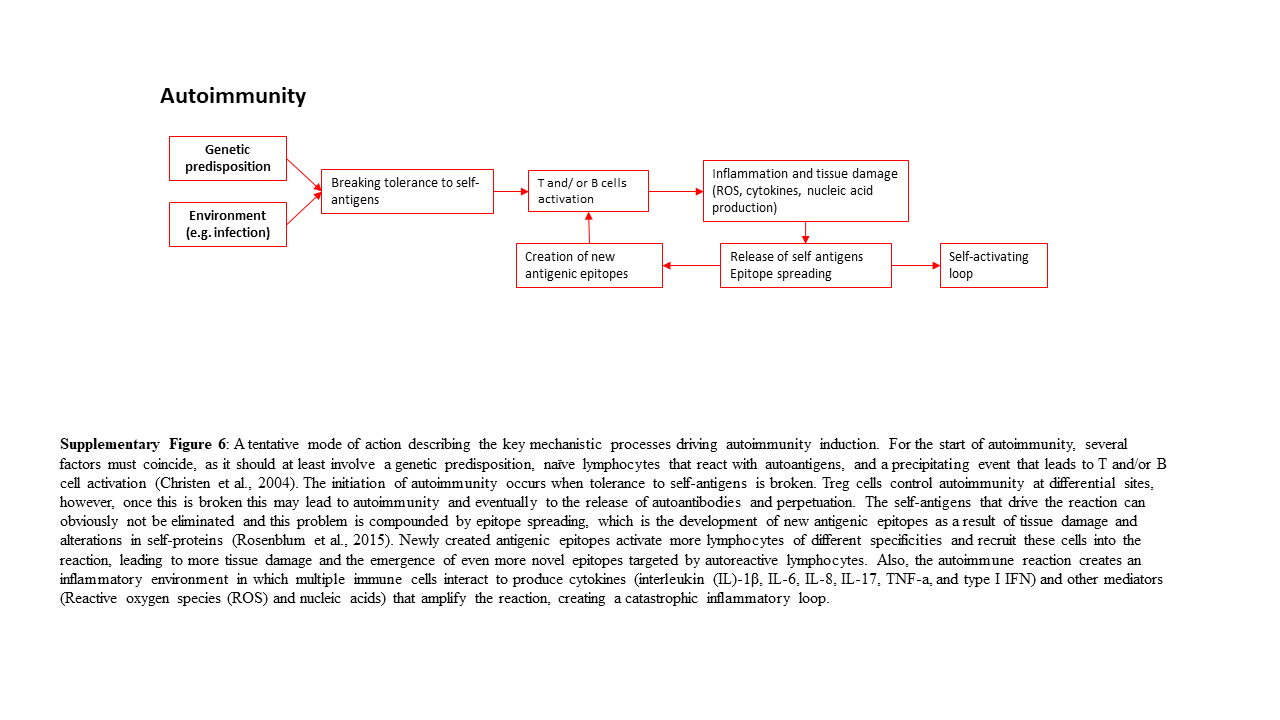

Supplement: Supplementary file 10 [file Image_6.tif]

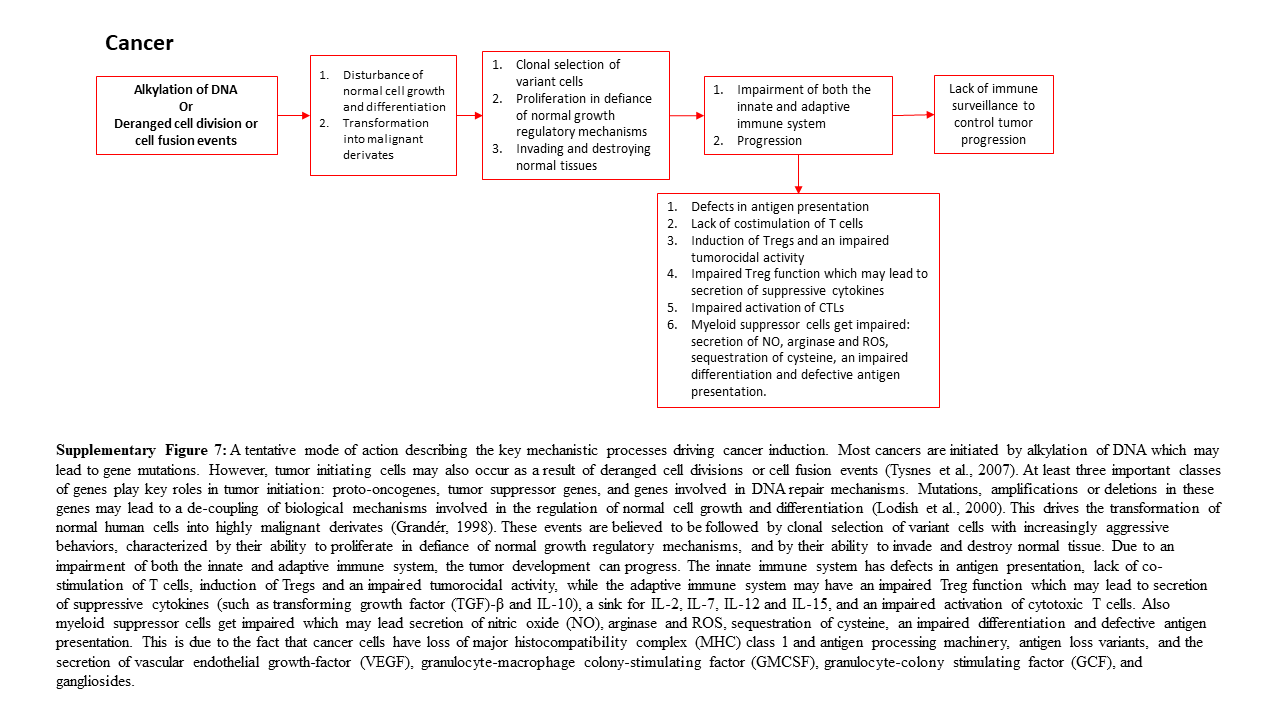

Supplement: Supplementary file 11 [file Image_7.tif]
